# Setting the stage: reviewing current knowledge on the health of New Zealand immigrants—an integrative review

**DOI:** 10.7717/peerj.5184

**Published:** 2018-08-23

**Authors:** Blessing Kanengoni, Sari Andajani-Sutjahjo, Eleanor Holroyd

**Affiliations:** 1 School of Public Health and Psychosocial Studies, Auckland University of Technology, Auckland, New Zealand; 2 Department of Nursing, Auckland University of Technology, Auckland, New Zealand; 3 Department of Nursing Research Capacity Development, Aga Khan University, Uganda

**Keywords:** Immigrant, Refugee, Healthcare, Health policy, New Zealand, Migrant, Health

## Abstract

The growth of migrant communities continues to rise globally, creating unique and complex health challenges. Literature on immigrant health in New Zealand (NZ) remains scant. This integrative literature review was conducted drawing on peer-reviewed research articles on immigrant health in NZ published between 2012 and 2018. The objectives were to: (i) provide a critical overview of immigrant health in NZ; (ii) identify general trends in health research conducted in NZ on immigrants; (iii) compare, contrast, and evaluate the quality of the information; (iv) develop a summary of research results and; (v) identify priorities and recommendations for future research. A search yielded more than 130 articles with 28 articles constituting the foundation of the review. This review is timely following the rapid increase in the scale, speed, and spread of immigration and its potential for changing NZ’s national health patterns and priorities. This integrative review led to the four primary conclusions. Firstly, migration in NZ is a gendered phenomenon, as there has been more women and girls arriving as migrants in NZ and being at risk of poor health in comparison with their male counterparts. Secondly, studies on infectious diseases take precedence over other health problems. Thirdly, research methodologies used to collect data may not be relevant to the cultural and traditional customs of the migrant populations. Furthermore, a number of research findings implemented have failed to meet the needs of NZ migrants. Lastly, policy initiatives are inclined more towards supporting health practitioners and lack a migrant centred approach.

**What is already known about this topic?** Despite NZ becoming more ethnically and linguistically diverse, there is limited literature on the health of migrants living in NZ.

**What this paper adds?** This integrative literature review provides a critical overview of refugee and migrant health in NZ through reviewing and critiquing the current literature available. This paper identifies research trends, the general health of migrants in NZ, recommendations that could inform future migrant and refugee health research and health policies and initiatives to ensure effective and relevant health service provision to migrants.

## Introduction

Population mobility is a key challenge for the 21st century health policy and decision makers (Jakab et al., 2017; Mladovsky et al., 2012; Zimmerman, Kiss & Hossain, 2011). The United Nations leading agency on migration report an increase in global migration, to an estimated 244 million people (or 3.3% of the world’s population) who have moved across their countries’ borders (International Organisation For Migration (IOM), 2018a). The decision to migrate is often related to important life transitions, such as obtaining higher education, family stream visas like family reunifications or relocating with family, starting work or getting married (United Nations Department of Economic and Social Affairs Population Division, 2017a). But for others, migration is a source of violence and disruption, particularly for those moving in an irregular manner like human trafficking (Global Migration Group, 2014). Of these global migrants, about 10% (22 million) are internally displaced people or often known as refugees, who were forced to leave their countries mostly due to political instability (IOM, 2018a).

However, according to the World Migrant Report, the main reason people migrate especially to high-income countries is to seek employment, making migrant workers to be the large majority of the world’s international migrants (IOM, 2018a). Economic migration is believed to benefit the receiving country. A 2017 report on the economic impact of immigration in New Zealand (NZ) found that each migrant contributes about $2,653 to the government, which is 15 times higher that of $172 contributed by NZ born individual. The migrant’s family and country also stands to benefit considerably. The remittances received by the sending country frequently include improvements in other dimensions of human development, such as an increase in school enrolment rates (IOM, 2018a). Other impacts of migration include the breaking down of social networks with Newbold, Watson & Ellaway (2015) reporting loss of relations between family and friends. However, such unprecedented demographic growth also comes with specific challenges for health service provisions in receiving countries.

**Figure 1:**
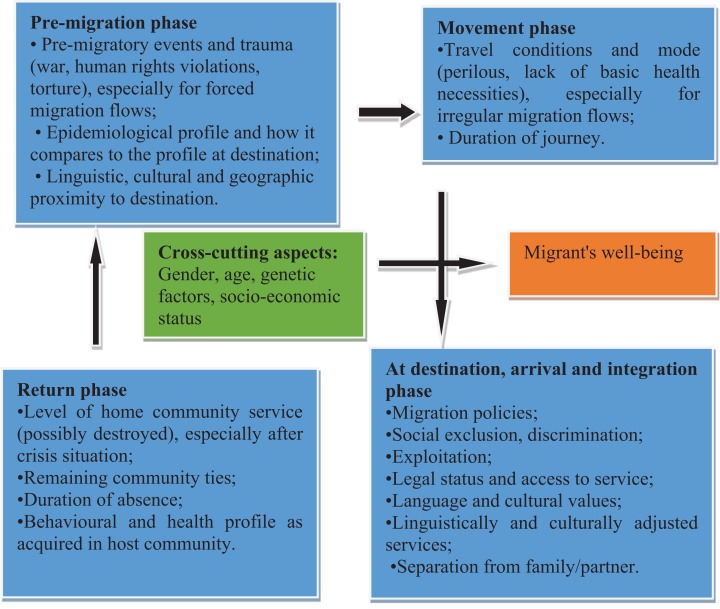
Social determinants of migrant health at all stages of the migration process (modified from IOM, 2018b).

The migrations process generally consist of four phases: ‘pre-migration phase’; the ‘movement phase’; the ‘arrival and integration phase’; and the ‘return phase.’ The determinants of migrants’ health can be identified at each stage (Fig. 1). The migration process is not a linear process, yet it may involve numerous and different entry points and may occur within or across national borders, exposing migrants and those they interact with to various risks that may make them vulnerable to physical and psychological problems (International Organisation For Migration, 2014; Simon et al., 2015; Zimmerman, Kiss & Hossain, 2011). Migrants’ vulnerability to ill health is increased by events that happened prior to arriving in a country; travel conditions; behavioural and health profile as acquired in host community and cross cutting aspects like gender as illustrated by the Fig. 1 below.

Immigrants are known to be generally in ‘good health’ at the time of moving to another country (Miramontes et al., 2015; Mladovsky et al., 2012). However, their health gradually deteriorates due to the interplay of various conditions that restricts their access to health services, including cultural and language barriers, lack of health insurance and knowledge of the health system (Babar et al., 2013; Harding et al., 2017; Miramontes et al., 2015; Pottie et al., 2014, 2017; Suphanchaimat et al., 2015). In some cases, it is the inability of the local health system to treat unfamiliar illness along with illnesses such as Human Immunodeficiency Virus (HIV) (Pottie et al., 2017), or the lack of training and skilled health professionals to deliver culturally sensitive services and to build rapport and trust (Shrestha-Ranjit et al., 2017). Furthermore, poor mental health has been observed as a salient feature amongst immigrants (Choummanivong, Poole & Cooper, 2014; Martinez et al., 2015; Reid, 2012; Shrestha-Ranjit et al., 2017), which may bring extra challenges to health professionals.

New Zealand is a culturally diverse nation, which requires relevant health policies and initiatives to work with this diverse population, including those on refugee and migrant health. NZ’s estimated population currently sits at 4,793,700, and projected to be 5.8 million by 2038, an average annual increase of 1.1% (Statistics New Zealand, 2017). According to the last published 2013 Census, a quarter (25.2%) of NZ population are foreign-born, with Asia being the common region of birthplace (Statistics New Zealand, 2013). Major refugee groups currently being resettled in NZ are from Asia Pacific, Africa, Middle East, and Latin America (Ministry of Business Innovation and Employment, 2017a). In 2017 and the immediate years prior, 750 refugees as per the current quota have resettled in NZ, each year. In July 2018, the annual quota for refugees’ settlement in NZ increased to 1,000 individuals (Ministry of Business Innovation and Employment, 2017a). It is therefore, anticipated that multi-cultural societies will continue to grow, with NZ’s highest rate of migrant growth being from the Middle East, Latin American and African ethnic groups (MELAA), and the rate of this group being expected to triple by 2038 (Statistics New Zealand, 2017). However, the top five sending countries of Immigrants in NZ, in the order of the highest sending country, is England, followed by China, India, Australia, and South Africa (Immigration New Zealand, 2017), with the age ranges from 20 to 29 years. The attractive immigration policy for skilled migrant categories and international students for tertiary education has been the main streams in which the immigrants come to NZ (Akbari & MacDonald, 2014), with more women migrating to NZ than men (Immigration New Zealand, 2017). The growth of the Asian population, is predicted to surpass the indigenous Māori population and likely to exceed one million by late 2020s. The annual growth rate of European or Other ethnic groups would be the lowest of 0.5 percent between 2013 and 2038 (Statistics New Zealand, 2017). These projections, therefore warrant steps towards proactively developing health strategies for migrant population.

It would be expected for countries like NZ to have specific migrant health policies which go beyond merely giving access to health care according to migrant status to adopting migrant health policies from countries like Ireland, which have ‘anti-discrimination and intercultural health care.’ According to Maldosky and colleagues (2012) this is perhaps the most balanced approach covering travellers, other ethnic minorities and children of migrants born in Ireland, in addition to migrants (including asylum-seekers, refugees, and undocumented migrants). Secondary analysis of published data may be useful in providing a knowledge base of refugee and migrant health in NZ and existing gaps to inform future direction of migrant health research in NZ. Gaps in research will be highlighted through the following objectives: (i) to provide an overview of migrant health studies ever conducted in NZ which are published in peer-reviewed journals, between 2012 and 2018; (ii) to identify general trends in health research conducted in NZ on immigrants; (iii) to compare, contrast and evaluate quality of the information; (iv) to develop a summary of research results and; (v) to identify priorities and recommendations for future research.

## Methods

Several strategies on collecting data were employed to compliment the evidence sought from a relatively limited body of literature on refugee and migrant health in NZ. The proposed strategies included the use of three modes of literature searches, using informal, primary and secondary modes. The informal mode included making connection with relevant researchers within the authors’ networks on migrant and refugee health. The primary mode included the traditional way of searching for articles in reference listings; and the secondary mode included search of relevant government documents (Auckland District Health Boards strategic planning documents, annual plans and related publications; and funded initiatives with a key focus on migration related research by Health Research Council of New Zealand) and reference databases. A total of four databases were used to search relevant papers: Sciencedirect, CINAHL, PubMed, and Scopus. These databases provided access to large databases of peer-reviewed scholarly literature. The policy documents assessed were restricted to those of the Auckland region as Auckland hosts 60 percent of migrants living in NZ (Gilbertson & Meares, 2013; Gray, Hilder & Stubbe, 2012). One hundred and thirty articles were extracted from the three search modes and 28 were used in this review. The inclusion criteria of selected texts were: (1) to have been published between 2012 and 2017; (2) to fall into the key words used for the search migrant health, refugee health, health policy, health service, health management and NZ; (3) articles that mentioned NZ in their studies. The nature and growth of migrant communities has drastically changed over the years, creating unique health challenges at a scale that has never been seen before. The authors therefore, believed that literature published before 2012 may not provide the true picture of the current trend of migrant health. The key words used for the search and in particular, the need for the studies to be NZ based enables the provision of a knowledge base of refugee and migrant health in NZ and existing gaps to inform future direction of migrant health research in NZ.

## Results

Table 1 provides a visual analysis of the points extracted from the articles using headings: author, title, topic, and type of research design, demographic characteristics of study population, limitations, findings, and recommendations. Thematic analysis was employed to come up with four themes. This analytical approach provided a suitable means to integrate qualitative and quantitative evidence.

**Table 1:** Studies by author, year, title, size, study population, objective, and methodology.

Reference	Size (*n*)	Study population	Objective or research question	Methodology
Gray, Hilder & Stubbe (2012)	Not mentioned	Not mentioned	To identify the actual pattern of use of interpreters for migrants and refugees	Qualitative Desk review
Rungan et al. (2013)	343	Under five children of refugee status from Africa, Asia, Middle East	To evaluate health needs of refugee children less than 5 years of age	Retrospective audit of outcomes of health screening data
Denholm, McBryde & Brown (2012)	Not mentioned	Current Australian and New Zealand immigration policies on TB; relevant Federal Government Dept websites and publications	To review the potential alternatives for the introduction of LTBI screening into Australian Immigration policy with an ethical lens	Qualitative Desk review
Shrestha-Ranjit et al. (2017)	40 (32 females, eight males) 12 healthcare providers (five nurses, four doctors, three midwives)	Bhutanese women and men; Health service providers	To examine the effectiveness of primary health care services in addressing mental health needs of Bhutanese refugee women resettled in New Zealand	Qualitative
Mette Fløe Hvass & Wejse (2017)	53	Study articles	To summarise the current literature on health screenings implemented after resettlement, regarding the content of the screenings and how they may differ across countries	Qualitative Systematic literature review
Douglas et al. (2017)	5	Immigration and Refugee Health Working Group countries: USA, New Zealand, Australia, UK and Canada	To describe the screening programmes, provide qualitative examples of capacity building that has occurred through those requirements and highlight how this capacity can be used to benefit broader management efforts	Qualitative Descriptive analysis of TB screening programmes
Jackson, Pinto & Pett (2014)	Not mentioned	Epidemiological and clinical evidence and policy documents Health experts in Australia and New Zealand	To investigate evidence, practices and policies pertaining to Chagas disease in Australia and New Zealand	Quantitative Qualitative
Martinez et al. (2015)	40	Policy documents	To assess and understand how immigration policies and laws may affect both access to health services and health outcomes among undocumented immigrants	Quantitative Qualitative
Babar et al. (2013)	11	Four Chinese, seven Indians	To explore attitudes, beliefs and perceptions of a cohort of migrants about medicines access and use in New Zealand	Qualitative
Browne et al. (2014)	104	Patients, health service providers, health policy makes within diverse clinical contexts	To examine the potential quality, utility and relevance of ethnicity data collected at an organisational level as a means of addressing health and healthcare inequities	Qualitative
Benkhalti et al. (2015)	117 HIAs and two HIA evaluations	Health Impact Assessments	To map out the extent and nature of the inclusion of migrants in HIAs	Quantitative
Morgan (2012)	Not mentioned	Not mentioned	To examine the current implementation of HIA in New Zealand	Not mentioned
Pareek et al. (2016)	Not mentioned	Not mentioned	To discuss the impact of migration on the epidemiology of TB in low burden countries	Qualitative
European Centre for Disease Prevention and Control (2014)	28 EU/EEA countries	The European Surveillance System 28 EU/EEA countries	To collect relevant data not reported by EU/EEA countries	Quantitative
Denholm & McBryde (2014)	Not mentioned	Not mentioned	To evaluate the feasibility of eliminating TB as a public health issue in a low-prevalence setting with immigration-related strategies directed at latent tuberculosis	Quantitative
Wickramage & Mosca (2014)	N/A	N/A	To assess if Migration Health Assessments are a Mechanism for Global Public Health Good	Not mentioned
Choummanivong, Poole & Cooper (2014)	61	Burma/Myanmar, Cambodia and Laos, Somalia, Sudan, Afghanistan, Uganda, Rwanda, Assyrian Iraq and Arabian Iraq 20–70 years old Both sexes	To identify the perceived impacts of family reunification on resettlement outcomes, health and wellbeing	Qualitative
Marlowe, Bartley & Hibtit (2014)	1	The New Zealand Refugee Resettlement Strategy	To examine the strategy and its five main goals of self-sufficiency, participation, health and well-being, education and housing	Qualitative
Mladovsky et al. (2012)	19 Health experts 10 country reports	Health experts Country reports	To compare and contrast the content of this second level of migrant health policies, going beyond statutory entitlement, across Europe	Quantitative
White et al. (2017)	Australia, Canada, New Zealand, and the US, large immigrant- and refugee-receiving countries that comprise the Immigration and Refugee Health Working Group (IRHWG)	77,905TB cases, and 888 MDR TB	To identifying and compare immigration and distribution of foreign-born tuberculosis cases are for developing targeted and collaborative interventions	Quantitative
Henrickson et al. (2015)	703 (351 Females; 343 males) for quantitative 131 (76 females; 54 males) for focus group discussion	Resident Black African population in New Zealand, and Africans living with HIV	To explore HIV risks in Black African communities in New Zealand with a view to informing HIV infection prevention and health promotion programs	Quantitative Qualitative
Suphanchaimat et al. (2015)	37	Study articles	To systematically review the latest literature, which investigated perceptions and attitudes of healthcare providers in managing care for migrants, as well as examining the challenges and barriers faced in their practices	Quantitative
Birukila, Brunton & Dickson (2014)	245 participants (150 men and 95 women) with a mean age of 28 years (range 16–58)	13 African countries	To describe the demographic characteristics of, and HIV-related risk behaviours among, black African migrants and refugees in Christchurch	Quantitative Cross sectional survey
Came et al. (2016)	One NZ Health strategy	New Zealand Health strategy	To critique the strategy in as it relates to health equity particularly for Māori	Qualitative
Horner & Ameratunga (2012)	Not mentioned	2007 New Zealand’s Settlement National Action Plan and other relevant social policy documents	To discuss the 2007 New Zealand’s Settlement National Action Plan and other relevant social policy documents in view of non-existent data and the politicised nature of immigrant research	Qualitative
Harding et al. (2017)	14	General practitioners and registrars	To identify assess the needs and attitudes of GPs in treating refugees and the perceived effect that refugees have on their practice	Qualitative
Gilbertson & Meares (2013)	Not mentioned	Health Service Providers from different ethnic groups, backgrounds, professions, activities and localities	To provide the following information on Middle Eastern, Latin American and African populations residing in the Auckland region	Quantitative Qualitative

### Literature search

The search retrieved 130 articles. First titles and abstract of those 130 articles were reviewed by the three authors to determine their relevance, using the following inclusion criteria: (i) the study was conducted in NZ; (ii) the study was published between 2012 and 2018, and (iii) the study focused on some aspects of refugee and migrant health and wellbeing. There is a notably interchanging of the term ‘Asian’ and ‘immigrant’ in many health and well-being research and policy setting as if the two terms are related. Asian health, therefore, appears to dominate the current picture of immigrant health in NZ. The final 28 original research articles were included in this review.

The literature on NZ refugee groups appeared in one third of the 28 articles reviewed (32%; *n* = 9) (Choummanivong, Poole & Cooper, 2014; Harding et al., 2017; Marlowe, Bartley & Hibtit, 2014; Marlowe & Elliott, 2014; Mette Fløe Hvass & Wejse, 2017; Rungan et al., 2013; Shrestha-Ranjit et al., 2017; White et al., 2017). Studies that focus on migrants accounted for 25% (*n* = 7) (Babar et al., 2013; Benkhalti et al., 2015; Birukila, Brunton & Dickson, 2014; Gilbertson & Meares, 2013; Gray, Hilder & Stubbe, 2012; Henrickson et al., 2015). Six studies (21%) had health providers and experts as the study sample (European Centre for Disease Prevention and Control, 2014; Harding et al., 2017; Mladovsky et al., 2012; Perumal, 2011; Pottie et al., 2014, 2017; Ross et al., 2016). Only one study (3%) included both the migrants and health service providers in their sample (Browne et al., 2014).

### Health topics

About a third of the 28 articles (36%; *n* = 10) focused on tuberculosis (TB) (Carballo et al., 2017; Denholm, McBryde & Brown, 2012; European Centre for Disease Prevention and Control, 2014; Henrickson et al., 2015; Martinez et al., 2015; Mette Fløe Hvass & Wejse, 2017; Pareek et al., 2016; Rungan et al., 2013; Seedat, Hargreaves & Friedland, 2014; White et al., 2017). Four studies were on mental health (14%) (Choummanivong, Poole & Cooper, 2014; Martinez et al., 2015; Reid, 2012; Shrestha-Ranjit et al., 2017). Three studies (11%) focused on HIV/AIDS (Adelowo, Smythe & Nakhid, 2016; Birukila, Brunton & Dickson, 2014; Henrickson et al., 2015). The number of studies on Chagas were two (7%) (Jackson, Pinto & Pett, 2014; Rungan et al., 2013). Diabetes and cardiovascular diseases accounted for another two studies (7%) (Gilbertson & Meares, 2013; Mehta, 2012). The remaining three of the five studies focused on the effectiveness and advocacy on migrant access to primary health care (11%). The other two studies (7%) were on the general wellbeing and social participation of refugees (Choummanivong, Poole & Cooper, 2014; Marlowe, Bartley & Hibtit, 2014). Government related sectors’ and district health boards’ policies and strategies including the Refugee Resettlement Strategy do not provide adequate and accurate information on refugee and migrant health and health care utilisation in NZ (Marlowe, Bartley & Hibtit, 2014; Minister of Health, 2016; Ministry of Business Innovation and Employment, 2017b; Stephens, 2017). Migrant surveys have been conducted annually since 2009 focusing merely on the experiences of recently new immigrants (Ministry of Business Innovation and Employment, 2017b).

### Research designs

Nearly half of the studies (*n* = 12; 43%) were qualitative (Babar et al., 2013; Browne et al., 2014; Choummanivong, Poole & Cooper, 2014; Denholm, McBryde & Brown, 2012; Douglas et al., 2017; Gray, Hilder & Stubbe, 2012; Harding et al., 2017; Mette Fløe Hvass & Wejse, 2017; Morgan, 2012; Pareek et al., 2016; Shrestha-Ranjit et al., 2017; Wickramage & Mosca, 2014). Twelve of these qualitative studies included relatively a small sampling, between 11 and 89 participants and mostly focusing on the health provider’s perspectives on their health provision to migrants (European Centre for Disease Prevention and Control, 2014; Harding et al., 2017; Mladovsky et al., 2012; Perumal, 2011; Pottie et al., 2014, 2017; Ross et al., 2016). Eleven studies (40%) were quantitative (Birukila, Brunton & Dickson, 2014; Came et al., 2016; Denholm & McBryde, 2014; European Centre for Disease Prevention and Control, 2014; Horner & Ameratunga, 2012; Marlowe, Bartley & Hibtit, 2014; Marlowe & Elliott, 2014; Mladovsky et al., 2012; Rungan et al., 2013; Suphanchaimat et al., 2015; White et al., 2017). The remaining five studies (2%) were mixed method (Benkhalti et al., 2015; Gilbertson & Meares, 2013; Henrickson et al., 2015; Jackson, Pinto & Pett, 2014; Martinez et al., 2015). None of these 28 studies had incorporated participant driven research methodologies nor included a diverse stakeholder groups in their sampling. Two of these 28 studies were cross sectional (Birukila, Brunton & Dickson, 2014; Henrickson et al., 2015) and none of them had used longitudinal study design. No health models or theoretical framework were used in any of these 28 studies. Knowledge acquired on migrant and refugee health has been informed by westernised paradigms which does not capture the relevant cultural and tradition customs which play an important role in the well-being of none westernised health outcomes.

### Characteristics of the study population

Only one study (3%) focused on children under 5 years of age (Rungan et al., 2013). The majority of the studies revolved around both adult women and men, with more than a third of the selected studies focusing on female participants (*n* = 10; 36%). The age range of adult participants was from 20 to 70 years (Babar et al., 2013; Birukila, Brunton & Dickson, 2014; Browne et al., 2014; Choummanivong, Poole & Cooper, 2014; European Centre for Disease Prevention and Control, 2014; Harding et al., 2017; Henrickson et al., 2015; Jackson, Pinto & Pett, 2014; Mladovsky et al., 2012; Shrestha-Ranjit et al., 2017). No studies had either migrant youth or migrant elderly as their study population studies. About half of the studies (*n* = 12; 45%) mentioned details of the countries of the refugees population and 20% of the articles (*n* = 6) did not mention specific countries of the migrant groups being studied.

## Discussion

The review proposes four primary conclusions. Firstly, migration in NZ is a gendered phenomenon when compared to men, women and girls have been seen as more vulnerable to reproductive health and mental health problems. Secondly, infectious diseases, especially TB and HIV/AIDS are seen as the most significant health problem affecting refugees and migrants. Third, knowledge acquired on migrant and refugee health has been informed by westernised paradigms which does not capture the relevant cultural and tradition customs which play an important role in the well-being of none western migrants. Forth, research on migrants has been biased towards the knowledge and perception of health professionals and none of the studies have employed a migrant centred study framework. Each finding has multiple implications for health policy and service delivery, discussed below.

### Migration and women’s health

Studies on migrant health, have been heavily focusing on female migrants or refugees and this may reflect to the current trend in global migration were females constitute 48.9% of women and girls of the total migrant population in developed countries (United Nations Department of Economic and Social Affairs Population Division, 2017b). For example, in Western Africa alone, the number of women migrating to European countries have significantly increased from 2.2 million in 1990 to 3.7 million in 2005, and 3.9 million in 2010 (Global Migration Group, 2014). Similar migrant trends have been observed in NZ by Callister & Didham (2012), with the exception of migrants from India. Unlike the Filipino migrants who supply more female nurses than males employment in the information, technology, and communication industries have been a pull factor for India male immigrants. A huge imbalance between Asian women and men living in NZ is seen with 26% more Asian women than men in the age group of 25–49 years, and 37% in the 30–34 years age group (Badkar et al., 2007). At the same time, NZ also offers a resettlement programme which gives priority to women and children (75 or more; 125 in 2016/17) who are considered to be marginalised and vulnerable (Immigration New Zealand, 2018).

Another aspect is that women’s and girls’ vulnerability to poor health is higher than their male counterparts. Their increased vulnerability is seen in sexual reproductive health where the Women’s Health Action organisation recognises sex and gender as important social determinants of health, which give rise to different health outcomes and health care needs between women and men (Women’s Health Action, 2015). In Australia, cultural incongruence has seen to have a significance influence in poor health seeking behaviour were migrants perceive the services to be inappropriate (Dune et al., 2017); same sentiment are echoed in NZ (Ministry of Social Development Summit, 2005). Mental health problems like emotional instability is also a common feature amongst young female immigrants (Choummanivong, Poole & Cooper, 2014; Martinez et al., 2015; Reid, 2012; Shrestha-Ranjit et al., 2017), where Suárez‐Orozco & Qin (2006) identify parenting practices which incite stress leading them to display more psychological symptoms than boys. Likewise, other international studies also document women being at high risk of occupational injuries (Dzúrová & Drbohlav, 2014; Moyce & Schenker, 2018).

### Heavy focus on communicable diseases, such as TB and HIV/AIDs

Studies on the health of migrants and refugees in NZ, like in many European and North American countries, rank TB as the number one infectious disease (Institute of Environmental Science and Research Ltd., 2015; Carballo et al., 2017; Denholm, McBryde & Brown, 2012; European Centre for Disease Prevention and Control, 2014; Henrickson et al., 2015; Martinez et al., 2015; Mette Fløe Hvass & Wejse, 2017; Pareek et al., 2016; Rungan et al., 2013; Seedat, Hargreaves & Friedland, 2014; White et al., 2017). It can be argued that most research funding has been allocated to control the spread of communicable diseases from developing countries to developed countries. Maternal deaths and morbidities for example, amongst other non-communicable diseases may been seen to affect only a small proportion of the population. However, studies on TB among migrant and refugees have been inconclusive. Others argue for the minimal risk of TB onward transmission from migrant as migrants and refugees to be screened for TB prior to entry (Douglas et al., 2017; European Centre for Disease Prevention and Control, 2014; Henrickson et al., 2015; Pareek et al., 2016; The Lancet Infectious, 2016; WHO, 2017). Others also argue for the fact that TB has never been fully eliminated from the European region and this should be addressed independently of TB risk associated with refugees (Centers for Disease Control and Prevention, 2017). Experience has also shown that when TB importation occurs, it is likely to involve regular travellers, tourists or health care workers rather than refugees or migrants (WHO, 2017).

Contrary to studies that suggest HIV to be prevalent amongst black African immigrants (Adelowo, Smythe & Nakhid, 2016; Birukila, Brunton & Dickson, 2014; Henrickson et al., 2015), the AIDS Epidemiology Group argue for some Africans to have been infected in NZ. Of the numbers of heterosexually acquired HIV cases in NZ, seven out of 19 people diagnosed were of African descent (AIDS Epidemology Group, 2015), suggesting that HIV is very much prevalent in other ethnic groups. Until recently, not much is known on HIV amongst immigrants in comparison with TB. Interestingly, the co-morbidity relationship between HIV/AIDS and TB has been well recognised since the beginning of the HIV epidemic and therefore, the prevalence of HIV infections should somewhat correlate with the TB prevalence. One possible explanation to this discrepancy may stem from the fact that HIV has recently been considered a notifiable disease in NZ in 2017 (Regional Public Health, 2017).

### Refugee and migrant mental health and non-communicable diseases

Mental health problems have been on the rise amongst NZ immigrants possibly due to anti-immigration policies as described by Martinez et al. (2015). Underutilisation of skilled migrants (Reid, 2012), lack of support system in resettling in NZ (Choummanivong, Poole & Cooper, 2014); lack of early detection of and awareness of mental health problems especially among refugees (Shrestha-Ranjit et al., 2017) accounts for the increase in mental health instability amongst foreign born nationals. Whilst migrating to a new country may ignite mental health challenges for refugees and migrants, we cannot rule out some benefits acquired as a result of migrating. For instance, refugees being in a ‘safe haven,’ away from their war conflicted countries, or voluntary migrants who migrant for an economic reward.

High prevalence of cardiovascular diseases and diabetes amongst the MELLA group (Gilbertson & Meares, 2013), have been noted in the NZ host community (Ministry of Health, 2014). The rising prevalence of diabetes and heart diseases in all three MELAA populations may indicate the acculturation effects of changes in diet, nutrition and physical activity that are associated with residence in NZ (Didham & Callister, 2012). This suggests the need for ethnic specific data and strategy formulations to the existence of transnational communities and the conditions for migrants along the continuum of the migratory process, rather than placing a singular focus on the migrants’ country of birth.

### Decolonising research methodologies in refugee and migrant health research

Research methodologies on immigrant health studies have been dominated by western conceptualisation of health and knowledge system. The application of western research paradigms often lead researchers to conduct research and draw conclusions that may not be relevant to the migrant or refugee population. Massey & Kirk (2015) argue for that the mismatched of analysing knowledge acquired within the Western scientific principles with the life context of and knowledge system of migrant and refugee communities. Applying distinct philosophies which are indigenous and cultural bound should not be complicated in culturally diverse countries like NZ. In the same light, the lack of enthusiasm for immigrant-driven or culturally-informed research design, and consequently, a lack of capacity in delivering relevant healthcare to immigrants also needs addressing.

### Policy initiatives are informed by health practitioners’ perceptions and not those of migrants

Policy initiatives typically involve training health workers, providing interpreter services and/or ‘cultural mediators,’ adapting organisational culture, improving data collection, and providing information to migrants on health problems and services. Challenges faced in NZ have included language barriers, time, culture constraints, and lack of information on services available for refers (Babar et al., 2013). Similarly to studies conducted in other receiving countries, health care providers frequently highlighted the need for additional support, interpretation services and or capacity building in delivering health care to migrants (Harding et al., 2017; Mladovsky et al., 2012; Ross et al., 2016). Today, policy initiatives continue to be informed by health experts and such initiatives have commenced research into improving data collection. Browne et al. (2014) found that health professionals in UK, USA, and NZ still perceive diseases to be distributed according to ethnicity, proxy for ‘racial’ classifications. This is in spite of extensive research showing that genetic differences account for only a very small proportion of the variation in disease patterns across so-called ‘racial’ groups and responses, with ethnic origin being a poor indicator of culturally-based practices, preferences or ethno-cultural identity. However, collecting individual-level ethnicity data stems from the assumption that such data reveals issues that contribute to health inequities, most notably, inequitable treatment, discrimination, or services that are poorly aligned with patients’ presumed ethno-cultural preferences. Some countries in the European Union (EU), therefore use the ‘country of birth’ variable as this is thought to be the most reliable indicator of whether or not an individual is a migrant (European Centre for Disease Prevention and Control, 2014). The country of origin also determines the level of assessment of possibly infectious diseases (European Centre for Disease Prevention and Control, 2014). However, it has shortfalls as it does not take into consideration when a migrant ceases to be a migrant or length of stay of migrant in country of birth before relocating.

Needless to say, one study in Australia, health practitioners propose the recruitment of international medical graduate doctors who may identify with resettlement problems faced by refugees and migrants (Harding et al., 2017). The study highlighted the awareness, empathy and positive attitudes of general practitioners in their approach to treating patients with migrant background. Such an approach further addresses specific health concerns unfamiliar to the NZ health providers who often are not trained to handle many of the health conditions immigrants present (Reiner, 2010). Perhaps recruitment of health professions of the same ethnicity and or culture may be one of the many ways to ensure adequate and quality service provisions for immigrants.

### Potential biases in the review process

There is a considerable possibility that articles dating before the year 2012 would have influenced the review in one way or another. However, due to the diversity and emancipation of modern migration, it is paramount that research be influenced by the current state of affairs. Lastly, the key word search method may have missed different terms referring to immigrants. Nonetheless, the technique used did include an extensive list of the most commonly used terms referring to refugees and migrants and such omissions are likely minimal.

### New research priorities

In summary, data on refugee and migrant health is highly fragmented and has an element of discrimination with an absence of longitudinal studies documenting changes in the health of this group. The following are proposed research priorities on refugee and migrant health based on the findings of this review:

1. Further studies on TB. Studies on TB and the vessel of TB transmission in receiving countries is inconclusive. Children of voluntary migrants under the age of 11 are not screened for TB, yet children that age are not immune to TB. Returning residents, regular travellers and those with visas that do not require TB examinations are not subjected to TB screening. Studies should therefore assess the magnitude of TB amongst these group of people. This may inform screening policy changes for the people coming into NZ.
2. More rigorous study designs. Studies that incorporate participant driven research methodologies should be embraced more. What is more important is to use distinct philosophies in which the target group identifies with. The traditional knowledge and how it is acquired provides understanding and is a basis for problem solving to effect change in health care. In addition, although larger studies such as longitudinal studies are known to have setbacks which include high attrition rates of participants lost to follow ups, larger funded quantitative and/or qualitative studies are needed to be able to assess changes of immigrant health over time.
3. Research on health needs of immigrant vulnerable groups. Studies on vulnerable groups like migrant youth, women, old age, and immigrants with disabilities did not feature in this review. It is such groups who require more health interventions and are at the receiving end of poor access and utilisation of health services.

## Conclusion

An integrative review of peer reviewed research articles shows female-dominated migration but more needs to be known about the general drivers of gendered migration to NZ, although there is evidence to conclude social and economic conditions in the country of origin as well as social and economic circumstances in NZ are some of the reasons. Sex and gender are also important social determinants of women’s health. Studies on onward transmission of TB from immigrants to host population are inconclusive. However, TB is most likely to involve regular travellers, health care workers than refugees and migrants. A further analysis of diseases distribution in NZ show immigrants to have similar health conditions to those in the host community. Therefore, sound prevention strategies that pay attention to the existence of transnational communities and the conditions for migrants along the continuum of the migratory process is key, rather than placing a singular focus on country of birth. Western research paradigms often lead researchers to conduct research and draw conclusions that may not be relevant to the migrant or refugee population. NZ migrant health policies may stand to be relevant to its beneficiaries if they are informed by studies that use distinct philosophies that recognise the importance of Indigenous knowledge and the cultural and traditional aspects that play a role in the health and well-being of migrant individuals. Policy initiatives continue to look for ways to assist health experts in providing optimum health services yet recruitment of international healthcare providers that identify with refugees’ and migrants’ traditional and cultural backgrounds in addition to settling in a new country may partly be the solution.

## Supplemental Information

10.7717/peerj.5184/supp-1 Supplemental Information 1 Raw data of articles used. Articles by author, year, title, size, study population, objective and methodology.

## Additional Information and Declarations

### Data Availability

The following information was supplied regarding data availability: The raw data are provided in a Supplemental File.
