# Supplementary material for: Setting the stage: reviewing current knowledge on the health of New Zealand immigrants—an integrative review
Source: PeerJ. 2018 Aug 23;6:e5184. doi: 10.7717/peerj.5184 (PMC6109585; doi:10.7717/peerj.5184)
Supplement: Supplemental Information 1 — Articles by author, year, title, size, study population, objective and methodology. [file peerj-06-5184-s001.docx]

| **Author, yr, title** | **Type of document** | **Methodology Characteristics of the study** | **Assessing methodological rigour** | **Discussion** | **Analysis of the reviewer** |
| --- | --- | --- | --- | --- | --- |
| (Gray, Hilder, & Stubbe, 2012)  "How to use interpreters in general practice: the development of a New Zealand toolkit." Journal of Primary Health Care **4**(1): 52-61{Gray, 2012 #139}{Gray, 2012 #139}{Gray, 2012 #139} | Journal article | **1.Non- Research**   - Literature review   2. **Objective or research question**  To identify the actual pattern of use of interpreters for migrants and refugees  **3.Sample:**  Not mentioned  4. **Methods**  Qualitative Desk review | No detail approach of methods used.  Study does not quantify how many policies of how many DHBs were reviewed | - 1. **Results**   There is gross underuse of interpreters  1.2. **Recommendation**  To evaluate efficacy and acceptability of the tool | Violation of rights to comprehensive healthcare due to no consistent use of interpreters in Nz.  It is not clear the ethnicity groups of the trained (Gray et al., 2012; Horner & Ameratunga, 2012; Suphanchaimat, Kantamaturapoj, Putthasri, & Prakongsai, 2015; White et al., 2017) and what measure was used to identify and train the particular interpreters.  The developed NZ toolkit was informed by the health practitioners. Patients were not part of the consultation.    Overall, the toolkit is not migrant health sensitive and lacks a rights based approach |

| **Author, yr, title** | **Type of article** | **Methodology Characteristics of the study** | **Assessing methodological rigour** | **Discussion** | **Analysis of the reviewer** |
| --- | --- | --- | --- | --- | --- |
| (Rungan, Reeve, Reed, & Voss, 2013)  Health Needs of Refugee Children Younger Than 5 Years Arriving in New Zealand." The Pediatric Infectious Disease Journal 32(12): e432-e436. | Journal article | 1.**Research**  Quantitative  2**. Objective or research question**  To evaluate health needs of refugee children less than 5yrs of age  3. **Sample**  **3.1 Type of participants**  Children of refugee status  **3.2 Size**  343  **3.3 Characteristics**  • Under 5  • Female and male  • Africa, Asia, Middle East  4. **Methods**  Retrospective audit of outcomes of health screening data | Researchers adheres to the prescription of the chosen methodology | - 1. **Results**   343 refugee children U5 who arrived in Nz between 01/01/07 and 31/12/12 represented 10% of the total population seen at the Mangere Refugee Resettlement Centre.  Even distribution of sex: 51% females, 49% male.  Age ranged from 7 wks to 4yrs 51 wks.  72% were from countries under the GAVI program.  Largest group from Asia at 53%.  Regional proportion of refugees similar with worldwide refugee population although this study had no refugee from Europe.  All children received informal verbal development screening.  96% required an interpreter.  No active TB cases  86% treated for Latent TB on the basis of positive Tuberculin skin test (TST) with 16% from Africa, 24% America, Asia 24% and ME 18%.  58% had the parasite giardia varying between regions; 16% had ascarias and 14% trichuris  66% received a complete vaccination certificate  73% needed one or more vaccines to align them to the NZ vaccination schedule   - 1. **Study Recommendation**   Screening of malaria and Helicobacter pylori at the MRRC as is the international standards. Such diseases have been found to be common amongst refugees is not currently screened for. | TST normally presents false positive results in subjects that have received a BCG vaccine. The refugee centre carries out TST on children that have no report of receiving BCG basing it on verbal and documentations, which are not reliable. Therefore the 86% cases presenting Latent TB can comprise of false positive results, who will receive medication that is quite detrimental to their health. It is therefore suggested that Interferon-gamma release assays (IGRA), though expensive will address this.  Countries have different vaccination schedules therefore the high number that received vaccination does not necessarily equate to a health need that was not met in the country of origin. This conclusion is supported by this study where it reports that 72% of the children came from countries under the GAVI program.  The study shows that a comprehensive health assessment is carried out on infectious diseases and follow on health services implemented. Though not part of the study, it is assumed that adults go through the same process. It is against this background that one can safely conclude that refugees are not transporters of infectious diseases but other types of migrants who are not subjected to such medical assessments. For instance asylum seekers (waiting for residence permits) only have limited access to healthcare and can only go through the medical examination when given refugee status, whilst transmitting the infection. In other cases, children of voluntary migrants under the age of 11 are not screened for TB, yet children that age are not immune to TB. Therefore there are perceived to be carriers of infectious diseases.  **Recommendation**  Further studies on health needs of refugee children from Europe to assess magnitude of their health needs in comparison with children from other regions. This may give a picture of the health systems in Europe and perhaps inform screening policy changes for the migrants coming from Europe |
|  |  |  |  |  |  |
|  |  |  |  |  |  |

| **Author, yr, title** | **Type of article** | **Methodology Characteristics of the study** | **Assessing methodological rigour** | **Discussion** | **Analysis of the reviewer** |
| --- | --- | --- | --- | --- | --- |
| (Denholm, McBryde, & Brown, 2012) | Journal article | 1.1 **Research**   - Qualitative   **1.2 Non- Research**   - Lit review of Current Australian and NZ immigration policies on TB; relevant Federal Government Dept websites and publications   2. **Objective or research question**  To review the potential alternatives for the introduction of LTBI screening into Australian Immigration policy with an ethical lense  4. **Methods**  Document analysis | Design of study correlates with conclusion. However, author should have stated the policies reviewed to give an overview of how many documents fed into the study. Further, stating the documents gives the readers the opportunity to review them and diagnose their own conclusion | - 1. **Results**   Applicants undergo a chest x-ray for testing for active TB prior immigration;  Children under 11 yrs and pregnant women are exempted from chest x-rays;  Follow up medical assessments may be required for those once infected by TB;  Justification of screening is to protect transmission of TB to host community and avoid burdening the health care system.  Potential Public Health policy changes may include:   1. **Tests used for screening**.   Tuberculin skin test has a record of producing false positive and falsely negative in setting of immune suppression or malnutrition. Interferon-gamma assays addresses the above but at a more expensive and technologically demand.  TB testing does not minimise burden on potential immigrants from an ethical point of view     1. **Circumstances for screening**   How and when it should be selected screening test be employed; in country of origin or post arrival screening in Aus or Nz although post arrival screening has been linked with lost to follow up.  There is an already stablished mechanism for Pre and post travel screening, a very effective procedure to identify TB positive cases, but would need to develop effectiveness for latent TB management  1.2 Recommendation | The TB screening tests currently used at the MRRC for under  5-year olds are inappropriate. TST has a high rate of false positive results which imposes unnecessary risk and burden on a child including risk of side effects from unnecessary treatment. More so, malnutrition has been a common feature amongst refugees. TST has been known to give false negative results in malnourished children.  Although health-economic analyses have shown that TB control in high income settings would benefit from providing targeted LTBI screening and treatment to certain migrants from high TB burden countries, implementation issues and barriers such as sub-optimal treatment completion will need to be addressed to ensure program efficacy. |
|  |  |  |  |  |  |
| Ethical evaluation of immigration screening policy for latent tuberculosis infection." Australian and New Zealand Journal of Public Health **36**(4): 325-328. |  |  |  |  |  |
|  |  |  |  |  |  |
|  |  |  |  |  |  |
|  |  |  |  |  |  |
|  |  |  |  |  |  |
|  |  |  |  |  |  |
|  |  |  |  |  |  |
|  |  |  |  |  |  |
|  |  |  |  |  |  |

| **Author, yr, title** | **Type of article** | **Methodology Characteristics of the study** | **Assessing methodological rigour** | **Discussion** | **Analysis of the reviewer** |
| --- | --- | --- | --- | --- | --- |
|  | Journal article | 1.1 **Research**  Qualitative  2. **Objective or research question**  To examine the effectiveness of primary health care services in addressing mental health needs of Bhutanese refugee women resettled in New Zealand.  **3. Sample**  **3.1 Type of participants**  Bhutanese women and men;  Health service providers.  **3.2 Characteristics**   - Above 18yrs - Both male and females - Bhutanese - Health providers: Doctors, nurses, midwives   **3.3 Size**  40 (32 women, 8 men)  12 healthcare providers (5 nurses, 4 doctors, 3 midwives)  4. **Methods**  5 FDGs with Bhutanese refugees  12 individual interviews with health professional | The researchers were explicit about how and why they choose specific design, thereby legitimizing criteria in ensuring the robustness of their inquiries | - 1. Results   32 Bhutanese women participated ranging from 18 to 82 years.  On average, the women had lived in refugee camps in Nepal for 18 years and in New Zealand for four years.  All women spoke Nepali language at home; however, more than half of them were unable to read and write in Nepali or to speak, read, and write in English, as they did not have any formal education.  Out of the 22 women who were engaged in occupations, 15 were working for their family without any payment and seven were employed in a paid job.  Most women followed Hindu religion, whereas a small number identified themselves as Buddhists and Christians.  Eight Bhutanese refugee men also participated, whose average age was 45 years, ranging from 26 to 55 years.  On average, they had lived in refugee camps in Nepal for 18 years and in New Zealand for five years.  All men spoke Nepali language at home and six of them had formal education, who could speak, read, and write in Nepali and in English. Four men were employed in a paid job and one was working for his family without pay.  Seven of them were Hindu and one was Christian.  Sources of mental issues were from Bhutanese participants:  Traumatic refugee journey;  Resettlement challenges;  family separation;  financial constraints;  lack of spiritual and social support;  Language, i.e. inability to communicate health needs;  Fragmented resettlement program which include job training, placement, English language lessons, and health literacy on mental health services  In total, 12 health professionals including four GPs, five nurses, and three midwives participated.  Among the 12 participants, all of the nurses (*n* = 5) and midwives (*n* = 3) were women and two of the four GPs were men.  On average, participants had 15 years of experience in their respective professions ranging from 2 to 43 years; five worked in private practices, three worked in semi-private (partly funded by the government), and four participants (the midwives and the public health nurse) worked at government funded practices.  Health service providers were concerned with:  language barrier compounded by lack of professional interpreter services;  need for cultural awareness and education;  time constraints associated with inadequate financial support from the government; and  need for a care package and an integrated family health centre.   - 1. **Recommendation**   Development of similar models of care to the Maori and Pasika for other cultural populations, such as refugees, in New Zealand.  Future research to explore Bhutanese refugee women's experiences related to gender discrimination and its impact on their mental wellbeing after they resettled in New Zealand and other host countries. | Mental health problems can be diagnosed amongst all different groups of migrants. Though not subjected to the same harsh travelling conditions of refugees, a typical immigrant can also go through financial constraints, lack of social and spiritual support, communication problems in the context of culture shock. Therefore, mental health policies should not aim to address mental health issues amongst all migrants.  It appears that the Refugee resettlement program falls short in building the capacity of refuges to be self-sufficient. It will be of interest to know if all resettlement programs are the same throughout Nz or there are specific to each district and/or region and if refugees in the other regions perceive the same as participants in this study.  PHO appear not to acknowledge refugee mental health, lacks focus and resources in meeting refugee mental health services. Moreso, the PHC has been reported to be fragmented not integrated.  Having a policy on interpreter services is one thing, and another on implementing it. It will be of importance to find out if the PHO in Palmerston North had been registered to receive the services at the time of this study or not |
| (Shrestha-Ranjit , Patterson, Manias, Payne, & Koziol-McLain, 2017)  "Effectiveness of Primary Health Care Services in Addressing Mental Health Needs of Minority Refugee Population in New Zealand." Mental Health Nursing **38**(4). |  |  |  |  |  |

| **Author, yr, title** | **Type of article** | **Methodology Characteristics of the study** | **Assessing methodological rigour** | **Discussion** | **Analysis of the reviewer** |
| --- | --- | --- | --- | --- | --- |
| (Mette Fløe Hvass & Wejse, 2017)  "Systematic health screening of refugees after resettlement in recipient countries: A scoping review." Annals of Human Biology **44**(5). | Journal article | 1.1 **Research**   - Qualitative   **1.2 Non- Research**   - Lit review   2. **Objective or research question**  To summarise the current literature on health screenings implemented after resettlement, regarding the content of the screenings and how they may differ across countries.  **3. Sample**  53 articles for review  4. **Methods**  Systematic literature review |  | - 1. **Results**   In spite of the heterogeneity, many of the studies included screening for the same diseases. Generally, there was a large proportion of studies focusing on infectious diseases. Only eight (15%) studies included screening for mental health.  Two studies included screening for chronic diseases (hypertension), although this is also a significant problem in refugee population.  There are considerable differences in the organisation of refugee screening programmes. The same refugee population from a certain country can be offered very different health screening programmes in their resettlement country, as these depend on the policy of the receiving country and not necessarily on the need of the individual.  In the US, the official Refugee Resettlement guideline suggests a ‘domestic screening’ of refugees within the first 90 days after arrival. This is in addition to the medical examination performed overseas  In New Zealand, refugees spend their first 6 weeks in a resettlement centre where medical screening is part of the programme  **1.2 Recommendation**  Further research is needed on health screening of the current refugee population regarding infectious diseases, non-infectious diseases and mental health.  An international consensus on how to perform and report studies on health screening in refugee populations where attention to non-communicable diseases is increased, including reporting of non-participating populations, would significantly increase the impact of future studies to the benefit of both host countries and refugees worldwide. | Consensus is yet to be reached on how, where and for whom screening should be implemented to be most effective.  Merit of screening is associated to the availability of diseases specific treatment programs, creating an ethical dilemma if screening is done at a time when treatment cannot be provided.  Policies appear to be more concerned with the welfare of its host population given the investment in screening of infectious diseases as opposed to chronic diseases like hypertension |

| **Author, yr, title** | **Type of article** | **Methodology Characteristics of the study** | **Assessing methodological rigour** | **Discussion** | **Analysis of the reviewer** |
| --- | --- | --- | --- | --- | --- |
| (Douglas et al., 2017)  Capacity strengthening through pre-migration tuberculosis screening programmes: IRHWG experiences. *The International Journal of Tuberculosis and Lung Disease, 21*(7), 737-745. | Journal article | 1.1 **Research**  Qualitative  **1.2 Non- Research**  Lit review  2. **Objective or research question**  To describe the screening programmes, provide qualitative examples of capacity building that has occurred through those requirements and highlight how this capacity can be used to benefit broader management efforts  **3. Sample**  **3.1** Immigration and Refugee Health Working Group countries: USA, NZ, AUSTRALIA, UK and CANADA  **3.2 Size**  5 countries  **3.3 Characteristics**  4. **Methods**  Descriptive analysis of TB screening programmes |  | - 1. **Results**   All five countries have pre-migration screening programmes for TB that are mandated through legislation.  The purpose of these programmes is similar—to prevent the importation of certain communicable diseases.  All five countries screen for infectious diseases.  Canada requires its panel physicians to adopt standards set by the national tuberculosis programmes (NTPs) in each country, augmented with WHO TB treatment recommendations and the latest Canadian standards  Australia and the USA also have a requirement to screen for latent tuberculous infection (LTBI), in which children aged 2–11 years in Australia or 2–14 years in the United States undergo a tuberculin skin test (TST) or an interferon-gamma release assay (IGRA) if they are examined in a country with an elevated rate of TB (greater than 40 per 100 000 for Australia, greater than 20 per 100 000 for the United States); treatment for LTBI is provided after arrival in the receiving country. For Australia, Canada and New Zealand, there is also a legislative requirement to avoid excessive health system costs  Four of the five countries provide panel physicians with their individual Technical Instructions, which stipulate how the examination should be performed. Canada requires its panel physicians to adopt standards set by the national tuberculosis programmes (NTPs) in each country, augmented with WHO TB treatment recommendations and the latest Canadian standards  Not all migrants are screened for TB. Policies vary among the different countries, balancing the need to protect public health and the practicalities of screening all individuals considered to have a high TB risk. Other considerations in developing screening policies include the duration and purpose of the visit and concerns that the cost of screening may act as a barrier to those seeking entry. Australia, Canada, New Zealand and the United States screen all refugees relocating to their countries and all permanent migrants, irrespective of TB incidence in the country of origin. Australia, Canada and New Zealand also undertake pre-migration screening for those coming for temporary stays of 6 months or more from countries with a WHO-estimated TB incidence of greater than 40/100 000. The United Kingdom screens all refugees relocating to its country, all permanent migrants and those coming for temporary stays of 6 months or more from countries with a WHO estimated TB incidence of greater than 40/100 000.  All five countries now have TB screening requirements that include a culture-based algorithm for TB disease screening.  The effectiveness of pre-migration screening has also been demonstrated with respect to the detection of drug-resistant TB, which would not be detected in the absence of rigorous screening programmes relying on culture and DST.  Capacity building:  building on existing infrastructure;  to develop programmes in countries of origin as part of a broader aid strategy or to deliver completely new infrastructure to support the sustainability of the screening programmes;  to build partnerships in-country and engage in strengthening NTPs.  Activities will include:  Increasing in laboratory capacity;  Greater individualised treatment and DOT;  Training and education of panel site personnel  **1.2 Recommendation**  Based on the evidence, it is recommended that panel physicians build relationships with the NTPs in their countries and explore opportunities for further collaboration to improve TB diagnosis and treatment in source populations. | It is important to mention that while some developing nations are struggling with the financial crisis, income per capita has been on the rise in China, India, and Brazil, and these quickly developing nations are experiencing a new flow of migration.  Further, migrants are generally known to be healthy and are able to afford to migrant. This may reflect a high quality of life which may not expose them to conditions favourable to TB infections. Therefore, it will be interesting to know TB incidences of this type of migrants from high risk countries in comparison with those from low risk countries. In the event that evidence shows no differences in TB incidences or shows a higher incidence of migrants from low risk countries, they may be a turn around on how policies are formulated  The study does not report on the categorization of the type of migrants and their respective proportion of TB cases identified  The group is not a legally constituted body, but rather a consultative forum that seeks to enhance the health security of migrants and receiving countries, the health services provided to migrants, and tuberculosis (TB) prevention and care globally. This means that are not legally obligated to do carry out right based health screening programmes  Adopting standards set by the national tuberculosis programmes (NTPs) in each country, augmenting with WHO TB treatment recommendations is the best approach as TB notifications derived from IRWHG programme will be entered in the system, which would have been cases missed by the system thereby not showing the real magnitude of the TB incidence.  Studies show that some constraints in providing migrant health is due to the health care staff not knowledgeable of how to treat diseases that are prevalent in sending countries yet this study show that health staff in sending countries are have their capacities enriched in order to detect and treat TB. Such an anomaly should be addressed by having health professionals in receiving country being trained and educated to provide optimum health care.  Panel physicians in most countries, like in Zimbabwe, conduct medical examinations and comply with the requirements of the IRHWG countries, thereby operating somewhat independently of the health care systems of their countries. Thus, the increases in TB capacity only benefit the populations that are leaving the country.  The merit of screening is associated to the availability of diseases specific treatment programs, creating an ethical dilemma were many countries lack the capacity to diagnose and treat TB |
|  |  |  |  |  |  |
|  |  |  |  |  |  |
|  |  |  |  |  |  |
|  |  |  |  |  |  |
|  |  |  |  |  |  |
|  |  |  |  |  |  |
|  |  |  |  |  |  |
|  |  |  |  |  |  |
|  |  |  |  |  |  |
|  |  |  |  |  |  |

| **Author, yr, title** | **Type of article** | **Methodology Characteristics of the study** | **Assessing methodological rigour** | **Discussion** | **Analysis of the reviewer** |
| --- | --- | --- | --- | --- | --- |
| (Jackson, Pinto, & Pett, 2014)  "Chagas disease in Australia and New Zealand: risks and needs for public health interventions." Tropical Medicine & International Health **19**(2): 212-218. | Journal article | 1.1 **Research**  Quantitative  Qualitative  2. **Objective or research question**  To investigate evidence, practices and policies pertaining to Chagas disease in Australia and New Zealand.  **3. Sample**  **3.1 Type of participants**  **3.2 Size**  **3.3 Characteristics**  4. **Methods**  Review of epidemiological and clinical evidence and policy documents pertaining to Chagas disease in the Western Pacific region.  Interviews with health experts in Australia and New Zealand about the clinical management of the disease and of its risk management in blood banks. | No detailed description of documents reviewed  No report on the number of participants and their respective professional background and the type of method used to collect data to enable assessment of the value of contribution and how the findings match with the discussion, conclusion and recommendations | - 1. Results   No epidemiological study about the real burden of Chagas disease in Australia, New Zealand or any Western Pacific country.  A few non-endemic countries have policies and programs aiming at identifying cases and preventing transmission.  Spain, the UK, France and Switzerland test blood donors with risk factors. While no non-endemic country has a national policy about screening for congenital transmission, it is practised in several regions and local institutions in Spain, Italy and Switzerland, where populations at risk reside.  No country in the Western Pacific region has any health policy designed to systematically identify individuals at risk of T. cruzi infection.  There is no programme to screen pregnant women at risk, their children and the rest of the family in case of positive results as shown effective elsewhere  No national recommendation pertains to testing blood and organ donors or persons with current or foreseen immunosuppression at risk of reactivation of the infection.  None of the two anti-parasitic drugs is registered or available in both Australia and NZ. Currently, doctors have to order them through special access schemes, which results in long delays before being able to initiate treatment.  WHO advocates a two-pronged strategy to tackle Chagas disease emergence and transmission in non-endemic countries: Interruption of transmission and facilitating access to treatment for infected persons as early as possible to prevent complications.  The current strategy of identifying and discarding donors at risk in Australia and New Zealand is in line with guidelines from countries with low risk of transmission.  In the absence of evidence about the real prevalence of *T. cruzi infection* in pregnant women, the cost–benefit ratio of such interventions cannot be assessed and it might be argued that New Zealand may host a too limited number of women at risk to afford favourable ratios.  Criteria and strategies for screening pregnant women differ among non-endemic countries and need to be adapted to the local epidemiology.  Community-based studies conducted in Europe showed that major risk factors for infection, in addition to being born in Latin America, were Bolivian origin, a maternal infection with *T. cruzi*, and being older than 35 years.  WHO has recently recommended screening pregnant women of Latin American origin who were born or had lived in Latin America; had received a blood transfusion in Latin America; or have a mother who had lived in Latin America. The use of similar criteria has yielded good results in different European countries.  Programme monitoring and evaluation would allow for adjusting these criteria to the Australian and New Zealand setting.  Providing effective care in Australia and New Zealand will require at the community level, patients' mobilisation to raise awareness of the disease and raising awareness and education among health professionals in addition to ensuring the availability of tests and drugs  **1.2 Recommendation**  We believe that Australia and New Zealand should align with some European non-endemic countries in putting in place: (i) epidemiological studies to better understand the context and risks; (ii) selective screening programmes for persons at risk; (iii) awareness programmes for clinicians in different sectors likely to meet with persons at risk, including travel clinics; (iv) improved access to diagnostic tools and treatment; and (v) links with Latin American communities to share information and education materials | Chagas disease is an emerging health issue in Australia and New Zealand likely to increase in importance given the rapid rise of the population at risk.  Chagas disease receives only minimal public health attention, which is likely to have significant consequences.  The scarcity of data warrants using models whose assumptions, such as considering uniform and stable infection rates in each country of origin, have only limited accuracy.  It is likely that clinicians, through lack of familiarity, might not make the diagnosis even when faced with a patient with end-stage (Rungan et al., 2013) disease.  The absence of strategy to identify infected individuals – with the exception of blood donors in Australia and New Zealand – means that T. cruzi infection will further spread unnoticed, putting individuals and communities at risk of suffering avoidable morbidity with potentially important costs for society. |

| **Author, yr, title** | **Type of article** | **Methodology Characteristics of the study** | **Assessing methodological rigour** | **Discussion** | **Analysis of the reviewer** |
| --- | --- | --- | --- | --- | --- |
| (Martinez et al., 2015)  "Evaluating the Impact of Immigration Policies on Health Status Among Undocumented Immigrants: A Systematic Review." Journal of Immigrant and Minority Health **17**(3): 947-970. | Journal article | 1.1 **Research**  Quantitative  Qualitative  **1.2 Non- Research**  Systematic review  2. **Objective or research question**  To assess and understand how immigration policies and laws may affect both access to health services and health outcomes among undocumented immigrants.  **3. Sample**  **3.1** Policy documents  **3.2 Size**  40 critically appraised articles: 30 articles related to access to health services, and 10 related to health outcomes.  **3.3 Characteristics**  4. **Methods**  Policy analysis using a Multiple Stream model which explains how policies are made be national governments under conditions of ambiguity | Design of study correlates with conclusion. Author presented information on articles reviewed and the policies reviewed to give an overview of how many documents fed into the study.  The documents gave the readers the opportunity to review them and diagnose their own conclusion in comparison with the author’s conclusion | - 1. **Results**   Thirty critically appraised articles were included in this review.  Mixed-methods approaches have been used with the inclusion of both qualitative and quantitative approaches to assess the relationship between immigration policies and access to health services among undocumented immigrants. However, the use of focus groups and quantitative questionnaires to measure perceived discrimination and access to health services—along with the critical understanding of immigration laws through legislative reviews—seem to be the most appropriate mixed-method approach used  Immigration laws and policies explicitly provide or restrict access to health services. Three categories were identified regarding access to health services: (1) laws and policies restricting rights to access health services, (2) laws and policies granting minimum rights to health services, and (3) laws and policies granting more than minimum rights to health services.  Several laws prohibited or restricted immigrants from accessing basic health services, including emergency care. In particular, these policies explicitly stated that undocumented immigrants could not seek health services or contained clauses that prevented them from seeking health services and mandated professionals to report documentation status. Hence, being “undocumented” was used as a means of exclusion from vital services (e.g., HIV and STI services, prenatal care services) provided by governmental agencies or non-profit organizations receiving government funding  Some jurisdictions only provided health care to undocumented immigrants in detention centers. Other countries have explicit laws and policies in which undocumented immigrants are entitled only to emergency care or care specified in terms such as “immediate or urgent”  Anti-immigration policies were fuelled by an anti-illegal immigration rhetoric that came about as a result of “economic and social problems” in the country like in Australia, USA  Several studies have concluded that racism and discrimination are endemic in the delivery, administration, and planning of health care services.  A clear correlation was shown to exist between conditions in immigration detention centers and increased anxiety, depression, and overall stress.  Longitudinal research is needed to establish connections between immigration laws and physical health outcomes such as autism, hypertension, cardiovascular disease, low birth weight, and prematurity.  Not only are immigration policies identified as factors affecting the health outcome of immigrants, but also other social determinants including specific environmental conditions such as pollution and contamination of water, as well as pre-and-post migration experiences ranging from rape, sexual assault, and abuse to extortion and several other specific geopolitical and economic factors  Little is known about the most recent immigration policies across the world and their potential impact on services and health outcomes among undocumented immigrants.  Most TB patients (71 %) sought care for symptoms rather than as a result of the efforts of public health personnel to screen high-risk groups or to trace contacts of infectious persons  To combat lack of communication that inhibited healthcare access, policy makers should support efforts to provide health care workers with skills through appropriate language and cultural sensitivity workshops.  Concern about immigration status decreases the likelihood of receiving care.  **1.2 Recommendation**  Eliminating Discrimination in Health Care Settings:  1.Health care providers have an ethical and professional obligation to care for the sick. Immigration policies and laws should not interfere with the ethical obligation to provide care for all.    2.Health care providers should encourage and promote cultural diversity and linguistic competency training and education for health professionals, which should include awareness, respect, evidence-based research, and capacity-building components.    3.Health care providers should encourage and promote programs in continuing education at the local and national levels that assist health professionals in their efforts to better serve the needs of underserved populations.    4. Health care providers should build referral systems with other organizations in the community to provide better information to immigrants, in particular about life in the United States, their legal right, becoming a citizens, and educational opportunities.  The findings of this review corroborate the urgent need for a global call for action:  1.Global actors should push for countries to control the admission of people within its borders and to enact and implement laws designed to reduce unlawful entry, while also collaborating with border countries for effective, bilateral solutions.    2.Non-governmental organizations, hospitals, and clinics have an ethical and legal obligation under human rights laws to treat undocumented immigrants in emergency situations.    3.Society has a vested public health interest in ensuring that all residents have access to health care, particularly for communicable diseases.    4.Based on the need in almost every community for mental health services that are both affordable and culturally relevant, global health actors should promote health care systems that incorporate mental health services and integration of services. | Does the system of immigration detention in NZ adequately screen detainees for HIV and delivers a substandard level of medical care to those with HIV.  Are ARV treatments and PMTCT accessible to the non-citizens?  Do immigrants and refugees pay higher fees to access the reproductive health services (2) Once an immigrant or refugee is identified as HIV positive, are there further follow-ups on the patient such as detecting the immune status using CD4 count or testing the viral load (3) Do immigrants and refugees have referral rights to referral clinics/hospitals for follow-ups in case of certain health conditions.  The immigrants and refugees are required to enlist in the Medical Aids scheme which can help offset part of the costs for the desired services.  Are they asylum laws that medically repatriate undocumented immigrants?  Are they immigration laws that forbids issuing residence permits on medical grounds?  Anti-immigration policies and departmental policies with anti-immigrant rhetoric are a major global determinant of health, particularly mental health. |

| **Author, yr, title** | **Type of article** | **Methodology Characteristics of the study** | **Assessing methodological rigour** | **Discussion** | **Analysis of the reviewer** |
| --- | --- | --- | --- | --- | --- |
| (Babar, Pengelly, Scahill, Garg, & Shaw, 2013)  Migrant health in New Zealand: exploring issues concerning medicines access and use." Journal of Pharmaceutical Health Services Research **4**(1): 41-49. | Journal article | 1.1 **Research**   - Qualitative   2. **Objective or research question**  To explore attitudes, beliefs and perceptions of a cohort of migrants about medicines access and use in NZ.  **3. Sample**  **3.1 Type of participants**  Asian immigrants  **3.2 Size**  11  **3.3 Characteristics**   - 4 Chinese 7 Indians   4. **Methods**  Interviews  FGDs | The methodology identified and implemented in the study do address the issues of reliability. However, the methodology fails on issues concerning validity. Findings do not truly represent the phenomenon being measured. Quantitative methods may have yielded findings that truly address the problem statement | - 1. **Results**   Findings emerged to include themes from the interviews  across the following broad categories:  financial barriers: paying doctor and pharmacist, lack of affordability of over-the-counter medicines, sharing medicines with family and friends; (b) information transfer and knowledge of rules, systems and initiatives, particularly regarding subsidies and brand switching; (c) misconceptions due to culture and language barriers, including not understanding information and lack of compliance in symptom-free disease; (d) perceptions of high quality in prescription medicines; (e) non-disclosure of traditional medicine use and (f) variability of community pharmacy service provision, especially counselling.  1**.2 Recommendation**  Future research will need to quantify the extent of the issues and interventions should be developed and evaluated as ongoing research. | Migrants interviewed are only restricted to the Asian ethnic group, therefore findings may not be the views of other ethnic groups.  Larger quantitative and/or qualitative studies are needed in order to be able to generalise findings. |

| **Author, yr, title** | **Type of article** | **Methodology Characteristics of the study** | **Assessing methodological rigour** | **Discussion** | **Analysis of the reviewer** |
| --- | --- | --- | --- | --- | --- |
| (Perumal, 2011)  Health Needs Assessments for Middle East, Latin America and African people living in Auckland Region. Auckland District Health Board. Auckland. | Journal article | 1. **Research**   - Quantitative - Qualitative   2. **Objective or research question**  To provide the following information on Middle Eastern, Latin American and African populations residing in the Auckland region:  •demography  •socio-economic determinants of health  •population health status and health status inequalities  •patterns of health service utilisation and areas of unmet health needs  **3. Sample**  **3.1 Type of participants**  11 Health Service Providers from different ethnic groups, backgrounds, professions, activities and localities were chosen based on a list of interviewees  **3.2 Size**  11 Data sources for quantitative studies  11 semi-structured interviewees  4. **Methods**  An epidemiological analysis of the health status of MELAA populations in the Auckland region with relevant demographic information  A qualitative analysis of MELAA health needs by interviewing health service providers.  A thematic review on MELAA health needs from existing qualitative and quantitative consultation material and international literature  A stock take of services targeting the MELAA populations. | Design of study correlates with conclusion. Author presented information on articles reviewed and the policies reviewed to give an overview of how many documents fed into the study.  The documents gave the readers the opportunity to review them and diagnose their own conclusion in comparison with the author’s conclusion | - 1. **Results**   MELAA groups face significant barriers to accessing health care including: language and communication difficulties; health illiteracy in some groups; the cost of health care; a lack of cultural understanding by Health Service Providers; and poor understanding of the New Zealand health system, and, for some groups, of Western health care models.  Of the three groups. Middle Eastern peoples are the largest group in the Auckland region.  The report shows the need for targeted diabetes and cardiovascular disease preventive strategies. Better access to womens' health, and primary oral health services in adults and children is also a key priority for Middle Eastern groups.  African peoples are the second largest MELAA group in Auckland. The report highlights the need for targeted diabetes prevention strategies for African groups, along with improved access to screening services, womens' health services, and better access to oral health care.  Latin American peoples make up the smallest proportion of the MELAA group.  The rising prevalence of diabetes and heart disease in all three MELAA populations may indicate the acculturation effects of changes in diet, nutrition and physical activity that are associated with residence in New Zealand.  1.2 **Recommendation**  To give feedback and share the information with these communities post report completion and to then collaboratively formulate strategies to meet their health needs. | NZ census of 2014 note that CVD and Diabetes are prevalent amongst the Nz host community  No data source on TB was included in the study yet high TB incidences are noticeable feature amongst the refugees and migrants.  No HIV/AIDS incidences were reported from the MELLA group. This could mean that the incidence was too low to be considered or that the population in question was not aware of the HIV status |

| **Author, yr, title** | **Type of article** | | **Methodology Characteristics of the study** | | | **Assessing methodological rigour** | | **Discussion** | **Analysis of the reviewer** |
| --- | --- | --- | --- | --- | --- | --- | --- | --- | --- |
| (Browne, Varcoe, Wong, Smye, & Khan, 2014)  "Can ethnicity data collected at an organizational level be useful in addressing health and healthcare inequities?" Ethnicity & health **19**(2): 240-254. | Journal article | | 1.1 **Research**   - Qualitative   2. **Objective or research question**  To examine the potential quality, utility and relevance of ethnicity data collected at an organizational level as a means of addressing health and healthcare inequities.  **3. Sample**  **3.1 Type of participants**  Patients, health service providers, health policy makes within diverse clinical contexts  **3.2 Size**  104  **3.3 Characteristics**   - Canada   4. **Methods**  3 FGDs community leaders;  16 interviews with health care workers; 60 semi structured interviews with patients;10 in-depth interviews with health policy makers | | | Design of study correlates with conclusion. | | - 1. **Results**   It is assumed that collecting individual-level ethnicity data has the following advantages: (a) identify health and healthcare inequities, (b)implement prevention and intervention programs and accountability standards to foster greater equity and (c) facilitate the provision of culturally and linguistically appropriate healthcare  Collecting individual-level ethnicity data stems from the assumption that such data are required to reveal issues that contribute to health inequities, most notably, inequitable treatment, discrimination, or services that are poorly aligned with patients’ presumed ethno-cultural preferences  Responses regarding ethnic origin are poor indicators of culturally-based practices, preferences or ethno-cultural identity  Another prevalent justification for collecting individual-level ethnicity data is that knowledge of patients’ stated ethnicity is needed to determine risks for genetically-mediated diseases  **1.2 Recommendation**  Take action universally to decrease discrimination and stereotyping within organizations, and at the point of care.  Make better use of existing data  Develop ways of collecting organizational-level data on perceived discrimination in healthcare. | Studies show that such data, when collected, have not led to decreases in health inequities  or significant improvements in healthcare  Ethnicity is presumed to be a proxy for ‘‘race’’, with the assumption that diseases are distributed according to racial classifications. These assumptions persist despite extensive research showing that genetic differences account for only a very small proportion of the variation in disease patterns across so-called ‘‘racial’’ groups |
| **Author, yr, title** | | **Type of article** | | **Methodology Characteristics of the study** | **Assessing methodological rigour** | | **Discussion** | | **Analysis of the reviewer** |
| (Benkhalti Jandua, Canuto de Medeirosa, Bourgeaultb, & Tugwell, 2015)  The inclusion of migrants in health impact assessments: A scoping review." Environmental Impact Assessment Review **50**: 16-24. | | Journal article | | 1.1 **Research**  Lit review  2. **Objective or research question**  To map out the extent and nature of the inclusion of migrants in HIAs**.**  **3. Sample**  **3.1** HIAs  **3.2 Size**  117 HIAs and 2 HIA evaluations  4. **Methods**  Quantitative and Qualitative analysis | Methodology is deemed to be the most appropriate given its systematic nature and its use in several other scoping reviews exploring issues in health policy and impact assessment | | - 1. **Results**   Migrants in analysis: 2 HIAs included migrants throughout the entire HIA which included the needs assessment component), baseline conditions analysis (which included the community proﬁling component), during impact analysis, and in the recommendations.  Migrants as stakeholders:  15 HIAs included the analysis of migrants as part of the stakeholder engagement. This did not mean that migrants themselves were included as stakeholders. Indeed, most often (11HIAs), this consisted in 3rd party organizations speaking on behalf of migrants or experts mentioning concern for migrant health. Only 4HIAs included migrants themselves as stakeholders  Special considerations for migrants. There were 6 HIAs which speciﬁed using special methodological considerations to include migrants in the analysis. These considerations included one or more of the following:(1) the literature review explicitly searched for information on migrants, (2) stakeholders were explicitly asked about migrants,(3) ensured organizations representing migrants were included,(4) translation of documents and workshops or interviews, and(5) tailored approach including a component of cultural sensitivity  Migrants remain seldom included and analysed in HIAs in contexts in which they would be expected to be analysed. When migrants are included, they are unlikely to be mentioned in the recommendations for mitigation measures, which represent the crux of the HIA process. A recent review assessing the reporting of HIAs in Australia and New Zealand similarly found that minority groups are often acknowledged, but are not analysed clearly for the potential impacts they may incur and corresponding recommendations.  **1.2 Recommendation**  There is a need for HIA practitioners to consider the potential relevance of migrants more frequently and provide more explicit rationale for the decision to include or exclude migrants from the analysis.  When migrants are included and analysis is relevant, practitioners could also draft recommendations which explicitly consider migrants.  When stakeholders are engaged in the assessment and migrants are a relevant minority group in the population assessed, adequate resources should be allocated to the use of speciﬁc considerations to facilitate their inclusion such as translation, cultural brokers, or tailored workshops.  Lastly, practitioners using HIA frameworks to guide their process should be careful of remaining restricted to only considering those groups explicitly listed in the framework. Indeed, such speciﬁc lists often found in HIA frameworks may have the unexpected effect of omitting groups who might be disadvantaged in certain contexts even if they are not listed  In terms of the broader practice of HIA, there is a need for greater standardization of reporting. | | It is necessary to distinguish between the factors inﬂuencing health of different ethnic minority groups versus those affecting migrant groups. While these factors may overlap, they have different  ramiﬁcations which may in turn translate into different considerations for the analysis and recommendations of an HIA. The concept of ethnicity is complex but implies the presence of either “shared origins or social background; shared culture and traditions which are distinctive, maintained between generations and lead to a sense of identity and group-ness; and/or a common language or religious tradition. Thus, an ethnic group may be a minority without being a migrant group while still facing health inequities for reasons that are different from those faced by migrants. Conversely, migrants might face similar barriers to optimal health irrespective of their ethnic background |

| **Author, yr, title** | **Type of article** | **Methodology Characteristics of the study** | **Assessing methodological rigour** | **Discussion** | **Analysis of the reviewer** |
| --- | --- | --- | --- | --- | --- |
| (Morgan, 2012)  "Institutionalising health impact assessment:the New Zealand experience." Impact Assessment and Project Appraisal **26**(1): 2-16. | Journal article | 1.1 **Research**  2. **Objective or research question**  To examine the current implementation of HIA in Nz;  To consider the extent to which differing views of HIA have affected institutionalisation of the process in Nz;  To assess the longest term prospects for the effective use of HIA for policy and project level assessment in NZ | No detail approach of methods used.  Study does not state if data gathered was from the HIA documents or from participants. | - 1. Results   HIAs are currently being implemented at project level  Social and cultural determinants appear not to be very popular in HIAs  The standard HIA at project level differ across councils  There is need of training and education to improve standard procedures on implementing HIAs. Many people involved from the Min of Health Sector lack awareness on the links of HIA and wider impact assessments theories and practises, affecting the form of the HIA developed.  Government departments are reluctant to have Min of Health interfering with their polices and also to invest in building the capacity of staff on HIAs  HIA at project and strategy level still being developed in NZ  1.2 Recommendation | Whilst the article states its findings, it is difficult to comprehend the data gathered as the reviewer is not aware of the type of research design employed. |

| **Author, yr, title** | **Type of article** | **Methodology Characteristics of the study** | **Assessing methodological rigour** | **Discussion** | **Analysis of the reviewer** |
| --- | --- | --- | --- | --- | --- |
| (Pareek, Greenaway, Noori, Munoz, & Zenner, 2016)  The impact of migration on tuberculosis epidemiology and control in high-income countries: a review." BMC Medicine **14**(1): 48. | Journal article | **1.2 Non- Research**   - Lit review   **Objective or research question**  To discuss the impact of migration on the epidemiology of TB in low burden countries;  To describe the various screening strategies to address this issue, review the yield and cost-effectiveness of these programs;  To describe the gaps in knowledge as well as possible future solutions. | Methodology was not stated therefore making it difficult to assess its rigour | - 1. **Results**   Tuberculosis in high-income countries continues to be a cause of morbidity and mortality – particularly amongst individuals who have been born overseas in high TB burden, low-income countries and migrated to high-income countries.  High burden of disease in the foreign-born, migrant, population are primarily due to migration from high TB burden settings and the reactivation of remotely-acquired latent TB infection. As a consequence there is increasing focus on how best to enhance TB control through the coordinated screening of migrants for TB.  Whilst most countries focus on screening migrants for active TB, this has a relatively low yield on its own and it is likely that the most effective and cost-effective means of screening migrants for TB will comprise multiple, inter-linking elements: pre-arrival screening for active TB and targeted post arrival screening for LTBI in migrants from intermediate/high TB burden settings.  However, the programmatic implementation of migrant screening is potentially hampered by limited uptake, acceptance and completion of therapy.  **1.2 Recommendation**  There is an urgent need for further coordinated research in this area to inform future national and international guidance. | The criteria for which migrant groups are screened is highly variable between countries with migrants coming from high TB incidences being common amongst the countries.  The reasons for this remain unclear but it may reflect a lack of evidence in this area.  Previous work has shown that the current models of active TB screening have weaknesses including: individuals not completing the screening processes, limited yields for active disease and an inability to identify active TB occurring through LTBI reactivation  Data on LTBI screening effectiveness found that migrants dropped out at each step of the screening pathway highlighting the need for research into interventions to optimise the LTBI screening pathway. Maybe health promotion and increasing access to services by means of finances?  Many studies do not adjust incidence rates for travel back to countries of origin nor underlying medical comorbidities like HIV and diabetes and chronic kidney disease. |

| **Author, yr, title** | **Type of article** | **Methodology Characteristics of the study** | **Assessing methodological rigour** | **Discussion** | **Analysis of the reviewer** |
| --- | --- | --- | --- | --- | --- |
| (European Centre for Disease Prevention and Control, 2014)  Assessing the burden of key infectious diseases affecting migrant populations in the EU/EEA. | Technical report | 1.1 **Research**   - Quantitative   2. **Objective or research question**  To collect relevant data not reported by EU/EEA countries to TESSy;  To identify literature not captured by the literature review;  To improve understanding of what data is available on infectious diseases among migrants in these countries;  To seek country views about the extent to which migrants are affected by infectious diseases.  **3. Sample**  **3.1 Data source**  The European Surveillance System (TESSy).  • A literature review.  • A survey sent to EU/EEA countries  **3.2 Size**  4. **Methods**  3 open ended Questionnaires three questionnaires on hepatitis B and C, gonorrhoea and syphilis, and measles and rubella. | Methodology is consistent with the results | - 1. Results   Most HIV cases reported among migrants from subSaharan Africa. However, the predominant mode of transmission among migrants also depends on country or region of origin.  Although the majority of TB cases in Europe occur in individuals born in the region, TB is also a significant issue among migrant populations.  TB notification rates are higher in foreign-born than native-born populations in most EU/EEA countries and, although overall incidence is declining in the EU/EEA, the opposite is the case among migrants.  Data on gonorrhoea and syphilis disaggregated by migrant status are only available from a few countries. These data show that, in 2010 11% of gonorrhoea cases were in migrants and 50% were in non-migrants and that 7.3% of syphilis cases were in migrants and 55.4% in non-migrants.  Of the 39.1% cases of hepatitis reported, 52.6% were imported.  Of the 10 271 cases of measles reported through TESSy in 2013, only 2.7% were categorised as ‘imported’ and 0.3% as ‘import-related’. Reasons for measles outbreaks vary between countries but include inadequate vaccination coverage. Studies from some countries suggest that migrant children may be at higher risk because they are less likely to be vaccinated against measles than non-migrant children.  99% of reported cases of malaria are ‘imported’.  Although the disease is not systematically monitored by countries in the EU/EAA, the number of Chagas cases reported has increased in the last decade and available data suggests that prevalence rates are high enough to warrant concern. Spain, Italy, the Netherlands, the United Kingdom, Germany and France have the highest number of estimated cases in Europe.  1.2 Recommendation | The ‘country of birth’ variable is used whenever possible, as this is deemed to be the most reliable indicator of whether or not an individual is a migrant. However, it has shortfalls as it does not take into consideration when a migrant ceases to be a migrant or length of stay of migrant in country of birth before relocating |

| **Author, yr, title** | **Type of article** | **Methodology Characteristics of the study** | **Assessing methodological rigour** | **Discussion** | **Analysis of the reviewer** |
| --- | --- | --- | --- | --- | --- |
| (Denholm & McBryde, 2014)  "Can Australia eliminate TB? Modelling immigration strategies for reaching MDG targets in a low-transmission setting." | Journal article | 1.1 **Research**   - Quantitative   2. **Objective or research question**  To evaluate the feasibility of eliminating TB as a public health issue in a low-prevalence setting with immigration-related strategies directed at latent tuberculosis**.**  **3. Sample**  **3.1 Data source**  **3.2 Size**  4. **Methods**  Mathematical modelling approach | Methodology is consistent with the results | - 1. **Results**   International strategies for reducing tuberculosis incidence emphasise early effective treatment of active infection and interruption of transmission - key strategies in high-transmission regions;  In a low prevalence context such as Australia, the considerable bulk of new tuberculosis cases may arise from reactivation of latent tuberculosis infection (LTBI) rather than local transmission.  Traditional public health approaches remains important, expanding public health efforts along these lines is unlikely to lead to further reduction in tuberculosis incidence in these settings.  Increasing recognition of the importance of LTBI reactivation in low prevalence contexts has led a number of countries to consider or develop immigration screening programs for LTBI.  Strategies in Australia focused on using combinations of tuberculin skin testing (TST)  /interferon-gamma release assays (IGRA; both Quantiferon Gold and Tspot-TB) and/or treatment with isoniazid, rifampicin or isoniazid with rifapentine preventative therapies.  Without additional intervention, tuberculosis incidence was predicted to reach 34.5 cases/million by 2050.  Strategies involving the introduction of an available screening/treatment combination reduced TB incidence to between 16.9–23.8 cases/million, and required screening of 136–427 new arrivals for each case of TB prevented.  Limiting screening to higher incidence regions of origin was less effective but more efficient.  1.**2 Recommendation**  Immigration-focused strategies cannot achieve the 2050 MDG and alternative or complementary approaches are required. | One way of curbing the reactivation of latent tuberculosis infection (LTBI) is addressing the conditions that makes migrants vulnerable to infections; their socio- economics state.  Is developing an immigration screening programs for LTBI a strategy NZ is banking on? Will the strategy be implemented pre or post migration? |

| **Author, yr, title** | **Type of article** | **Methodology Characteristics of the study** | **Assessing methodological rigour** | **Discussion** | **Analysis of the reviewer** |
| --- | --- | --- | --- | --- | --- |
| (Wickramage & Mosca, 2014)  "Can Migration Health Assessments Become a Mechanism for Global Public Health Good?" International Journal of Environmental Research and Public Health **11**(10): 9954-9963. | Journal article | 1.1 Non- **Research**  Literature review  1.2R**esearch question**  Can Migration Health Assessments Become a Mechanism for Global Public Health Good?  **3. Sample**  **3.1 Data source**  n/a  **3.2 Size**  n/a  4. **Methods**  Document analysis | Methodology was not stated to allow for evaluation of it | - 1. **Results**   HAs are usually conducted as a measure to limit or prevent transmission of diseases of public health importance to their host populations; and to avert potential costs and burden on local health systems, especially for the treatment of chronic disease conditions;  There are diverse range of HA models across sixteen countries that differ across diagnostic protocols used, for example, to screen for tuberculosis (TB), the site of testing and the category of migrants to be tested;  Public health consequences on those failing the HA are difficult to assess, considering most authorities seldom publish data on potential migrants who have undergone screening, the types of disease conditions and follow-up or referral outcomes;  A feature of migrant HAs processors is that they often operate within a “vacuum”, with little or no formal linkage to the public health system of the country of origin.  Governments of destination countries emphasize partnerships with national health authorities for disease surveillance requirements (as per the country’s public health regulations) and ensuring treatment and referral plans for those prospective migrants deemed non-admissible based on health status.  **1.2 Recommendation**  Migrant-sensitive health policies are therefore needed to inform immigration and international recruitment policies. For instance, establishing information systems to evaluate the effectiveness of immigrant screening to allow for evidence-based adjustments of HA policies have been highlighted in the Netherlands | Health screening in respect to TB testing are usually not linked with the National TB Programme  In the context of TB screening, were a potential migrant is denied a visa on medical grounds will have no provision of adequate counselling, treatment and follow up, nor will there be any contact tracing employed as preventative measures, in support of Wickramage and Mosca (2014) contention, HAs will remain an “immigration functional requirement” which benefits the destination country, rather than providing a public health good.  Approaching HAs in a more collective and meaningful forms of partnership with national health systems, will likely increase public health benefits, where for instance in the case of hepatitis C, a case-management plan for the potential migrant may be activated involving health education, referral to local health services for treatment and linkage to relevant health promotion |

| **Author, yr, title** | **Type of article** | **Methodology Characteristics of the study** | **Assessing methodological rigour** | **Discussion** | **Analysis of the reviewer** |
| --- | --- | --- | --- | --- | --- |
| (Choummanivong, Poole, & Cooper, 2014)  "Refugee family reunification and mental health in resettlement." Kōtuitui: New Zealand Journal of Social Sciences Online **9**(2): 89-100. | Technical report | 1.1 **Research**  Qualitative  2. R**esearch question**  Research questions focused on the perceived impacts of family reunification on resettlement outcomes, health and wellbeing.  **3. Sample**  **Type of participants**  Burma/Myanmar, Cambodia and Laos, Somalia, Sudan,  Afghanistan, Uganda, Rwanda, Assyrian Iraq and Arabian Iraq.  **Age**  20-70yrs  **3.1 Data source**  **3.2 Size**  61 participants  4. **Methods**  13 focus groups with 46 participants  15 face-to-face structured interviews. | Methodology is consistent with the results.  However, the small number of participants representing some cultures may raise questions about the validity of such generalisations. Additionally, the disproportionate number of females generally, and Somali females in particular, may raise questions about the generalisability of the study's findings to the wider refugee population. The fact that participants from all ethnic groups reported similar themes, however, suggests that this problem is unlikely to have detracted significantly from the generalisability of the research findings. The fact that New Zealand accepts a relatively high number of women at risk and children in its refugee quota could be seen as an appropriate rationale for determining the gender composition of the research sample. | - 1. **Results**   The absence of family is the greatest impediment to successful resettlement.  Family reunification was perceived by participants to critically influence their adaptation and participation in the local community and their wellbeing in terms of health, employment, financial stability and education.  Participants described the mismatch between official Euro-centric definitions and their own cultural understandings of ‘family’ as a major stumbling block associated with their applications for family reunification. Participants generally subscribed to an extended family perspective, defined by kinship in its widest sense.  Frustration with the difficulty, costs, length and complexity of the family reunification process was a very common theme prior to lodging their family reunification application, that the process would be relatively ‘easy’, ‘fast’ and ‘simple.  Concerns over family left behind and the emotional effects of separation.  Intergenerational conflict following family reunification  Unsuccessful reunification applications: mental health and resettlement difficulties  Community/family/local support as a positive contributor to resettlement  **1.2 Recommendation**  To ensure the provision of comprehensive, well-targeted support. To be effective, organisational processes and support must be culturally relevant and provided in a culturally appropriate manner.  A structured evaluation of the effectiveness of current social services and support programmes for families going through the family reunification process would now be timely.  Future research involving children and young people who have undergone family reunification would be particularly useful. Such research could explore the experiences and effects of family reunification on participants' health and wellbeing, social integration, schooling and academic achievement. | Whilst evidence show that refugees are more disadvantaged than the regular migrant, mental health issues and its linkages to absence of family also impedes successful resettlement of the migrant in the host country. Perhaps there should be further research on the comparison of mental health between the refugee and the regular migrant to ascertain the applicability of having mental health services delivery tailor made for refugees in the face of other migrants who may also require such services.  In a related study, there was noted of an association of poor mental health and under- utilisation of employment skills amongst skilled migrants (Reid, 2012) |

| **Author, yr, title** | **Type of article** | **Methodology Characteristics of the study** | **Assessing methodological rigour** | **Discussion** | **Analysis of the reviewer** |
| --- | --- | --- | --- | --- | --- |
| (J. M. Marlowe, Bartley, & Hibtit, 2014)  "The New Zealand Refugee Resettlement Strategy: implications for identity, acculturation and civic participation." Kotuitui: New Zealand Journal of Social Sciences **9**(2): 60-69. | Journal article | 1.1 **Research**   - Quantitative   2. **Objective or research question**  To examine the strategy and its five main goals of self-sufficiency, participation, health and well-being, education and housing  **3. Sample**  **3.1 Data source**  **3.2 Size**  4. **Methods** | Methodology is consistent with the results | - 1. **Results**   In 2012, the New Zealand government presented the New Zealand Refugee Resettlement Strategy with the overarching vision of refugees ‘participating fully and integrated socially and economically as soon as possible so that they are living independently, undertaking the same responsibilities and exercising the same rights as other New Zealanders and have a strong sense of belonging to their own community and to New Zealand. Within this vision statement, the strategy proposes five primary goals that are noted as ‘integration outcomes:  **Self-sufficiency**: all working-age refugees are in paid work or are supported by a family member in paid work.  **Participation**: refugees actively participate in New Zealand life and have a strong sense of belonging to New Zealand. Public receptivity to that diversity has not been universally positive, however, with persisting claims that newcomers to New Zealand remain vulnerable to discrimination  **Health and well-being**: refugees and their families enjoy healthy, safe and independent lives. The two primary goals within the health and well-being outcome are increased quota refugee children receiving age-appropriate immunisations (6 and 12 months after arrival), as well as increased utilisation of general practitioner services and increased access to mental health services. A key focus to achieving these goals is about addressing English language competencies and improving the health literacies of particular communities.  **Education:** refugees' English language skills enable them to participate in education and achieve qualifications, and support them to participate in daily life.  **Housing**: refugees live independently of government housing assistance in homes that are safe, secure, healthy and affordable.  **1.2 Recommendation**  To achieve ‘success’, then, the focus of government and non-government agencies must broaden beyond interfacing with and working alongside refugee-background individuals, families, groups and communities; their focus must incorporate the host society as well, to promote and mobilise wider social capital resources to inculcate the strong sense of belonging to which the strategy aspires. | The literature clearly demonstrates the challenges of acculturation and this strategy will need to be flexible in its approach to address the rich cultural, social, spiritual and historical backgrounds that refugees bring with them while also being holistic in its analysis around ‘outcomes’. |

| **Author, yr, title** | **Type of article** | **Methodology Characteristics of the study** | **Assessing methodological rigour** | **Discussion** | **Analysis of the reviewer** |
| --- | --- | --- | --- | --- | --- |
| (Mladovsky, Rechel, Ingleby, & McKee, 2012)  Responding to diversity: An exploratory study of migrant health policies in Europe." Health Policy **105**(1): 1-9. | Journal article | 1.1 **Research**   - Quantitative   2.**Objective or research question**  To compare and contrast the content of this second level of migrant health policies, going beyond statutory entitlement, across Europe.  **3. Sample**  **3.1 Data source**  Health experts  Country reports  **3.2 Size**  19 health experts  10 country reports.  4. **Methods**  The survey consisted of a questionnaire sent to each of the 19 health policy experts. The survey collected information on: collection and reporting of data on migrant health; government policies and programmes; and non-governmental policies and programmes. Under the section on government policies and programmes, the respondents were asked to report on the main elements of policies, programmes and/or legislation relating to health of or access to health care by migrants and/or ethnic minorities.  Document analysis of 10 country reports | Methodology is consistent with the results | Most of the national policies target either migrants or more established ethnic minorities.  Policies in several countries priorities specific diseases or conditions, but these differ and it is not clear whether they accurately reflect real differences in need among countries.  Policy initiatives typically involve training health workers, providing interpreter services and/or ‘cultural mediators’, adapting organizational culture, improving data collection and providing information to migrants on health problems and services.  Most European countries did not address migrants’ health and access to health services by means of specific policies: by 2009, only eleven of the 25 countries had established national policies that are aimed at improving migrant health and go beyond statutory or legal entitlements.  Most national policies simply use the word “migrant” (in the native language) to define the target population, but the nuances of exactly who is included and excluded are not specified. There is no established international definition of how much time must pass before a foreign national ceases to be regarded as a migrant, and when a migrant is considered to belong to a socially, culturally or ethnically distinct group. In general, the national policies focus either on migrants or on ethnic minorities, not both  A few countries stand out for their quest to increase migrants’ health literacy and their participation in the development and implementation of policy.  **Recommendation**  Targeting of specific diseases or conditions may in certain cases be somewhat arbitrary and should be revised to reflect the different health needs of migrants and deficiencies in existing health service structures.  Building on initiatives targeting patients and providers that are already in place, countries need to adopt more complex but possibly more effective approaches such as the “whole organization approach”.    Health literacy programmes and the participation of migrants in the development and implementation of policy should also be encouraged.  Finally, there is an urgent need for better monitoring and evaluation of policy implementation. | NZ has not addressed migrants’ health and access to health services by means of specific policies.  In the case of NZ, there is a notably interchanging of the term ‘Asian’ and ‘immigrant’ in many health and well-being research and policy setting as if the two terms are related. Asian health, therefore represents a picture of immigrant health.  Indeed, participation is an essential component of good governance but is rarely achieved in relation to vulnerable groups such as migrants.  Policy divergence across countries is not entirely surprising, given the different patterns and levels of immigration. For example Italy, Portugal and Spain have experienced large-scale immigration only relatively recently, so a focus on newly arrived migrants is understandable. However, new migrants face different health challenges to older migrants and the descendants of migrants.  The scarcity of data on implementation raises the concern that the adoption of national policies may not be felt on the ground.  Implementation is affected by a number of other factors, including the administrative arrangements in the respective country and its health system, demographic patterns of migration, election cycles, data availability, collaboration with other sectors, and budgetary restraints due to the current economic crisis.  The wide differences observed across (and sometimes even within) countries in the different dimensions of migrant health policies suggest that there are considerable opportunities for cross-country learning and policy dialogue. However, it is also important to recognise that countries have varying traditions and national contexts, so that there can be no “one size fits all” approach to migrant health policies in Europe. Distinguishing between “communitarian” and “republican” approaches to diversity might help explain why a country with a relatively long history of immigration such as France focuses narrowly on newly arrived migrants and has not yet developed intercultural or ethnic minority health policies. Further research is needed to understand how migrant health policies can be developed in different national contexts. |
| **Author, yr, title** | **Type of article** | **Methodology Characteristics of the study** | **Assessing methodological rigour** | **Discussion** | **Analysis of the reviewer** |
| (J. Marlowe & Elliott, 2014)  Global trends and refugee settlement in New Zealand. *Kōtuitui: New Zealand Journal of Social Sciences Online, 9*(2), 43-49. | Journal article | 1.1 **Research**   - Quantitative   2. **Objective or research question**  **3. Sample**  **3.1 Data source**  **3.2 Size**  4. **Methods** | Methodology is consistent with the results | - 1. **Results**   New Zealand’s human rights framework is central to the country’s refugee system.  Despite a nearly 70-year history of refugee resettlement, New Zealand does not have a formal refugee policy.  In response to the UNCHR Convection, in 2012, the New Zealand government launched the New Zealand Refugee Resettlement Strategy which is built on the principle that successful settlement is about willingness and capacity to participate fully in community and becoming ‘self-sufficient’.  Currently, there are only plans to implement the Refugee Resettlement Strategy in regards to quota refugees not those arriving as asylum seekers or under family reunification  1.2 Recommendation | Experiences of asylum seekers and convention refugees provides a critical understanding of a relatively under researched group in New Zealand. The different entitlements and rights that quota refugees and convention refugees receive provide a stark picture demonstrating that how one arrives as a refugee truly matters. |

| **Author, yr, title** | **Type of article** | | **Methodology Characteristics of the study** | **Assessing methodological rigour** | **Discussion** | **Analysis of the reviewer** |
| --- | --- | --- | --- | --- | --- | --- |
| (White et al., 2017)  Immigrant Arrival and Tuberculosis among Large Immigrant- and Refugee-Receiving Countries, 2005–2009. *Tuberculosis Research and Treatment, 2017*, 8567893. doi:10.1155/2017/8567893 | | Journal article | 1.1 **Research**   - Quantitative   2.**Objective or research question**  To identifying and compare immigration and distribution of foreign-born tuberculosis cases are for developing targeted and collaborative interventions.  **3. Sample**  **3.1 Data source**  Australia, Canada, NZ, and the US, large immigrant- and refugee-receiving countries that comprise the Immigration and Refugee Health Working Group (IRHWG).   - 1. **Size**   77,905TB cases, and 888 MDR TB  **3.3 Methods**  Data stratified by year and country of birth from 2005 to 2009 were received from five countries | Methodology is consistent with the results | - 1. Results   Data from the analysis reveal that India, China, Vietnam, and the Philippines supplied the majority of diagnosed  TB counts. These four countries accounted for combined adjusted 41.4% of the total foreign-born TB cases and 42.7% of the foreign-born MDRTB cases. Of these, India was the leading source country for arrivals (11.5%), TB cases (19.0%), and MDR TB cases from 2005 to 2009 (15.6%).  Data reveal that four of the IRHWG countries have a specific source country/ countries that contributes a significant amount of TB cases without affecting the group as a whole. Examples of specific source contributors include Papua New Guinea (3.4%of foreign-born TB cases and 42.4%of MDR TB cases) for Australia; Samoa (5.3% TB cases) for New Zealand; Somalia (10.6% TB cases and 12.3% MDR TB cases) and Pakistan (16.7% TB cases, 8.7% MDRTB cases) for the United Kingdom; and Mexico (24.1% TB cases and 13.7% MDR TB cases) for the United States. Canada received the majority of cases from the four largest source countries (India, the Philippines, China, and Vietnam)  **1.2 Recommendation**  A cost-effectiveness review of screening programs on potential collaborations | Australia, Canada, New Zealand, the United Kingdom,  and the United States are the largest immigrant- and refugee receiving countries in the world and are currently collaborating on preventing importation of TB into each of their countries. Joint efforts in a small number of high-burden countries can help prevent importation of TB cases and also contribute to control efforts within source countries.  Collaborative efforts across a small number of countries have the potential to yield sizeable gains in tuberculosis control for these large immigrant- and refugee-receiving countries. |

| **Author, yr, title** | **Type of article** | **Methodology Characteristics of the study** | **Assessing methodological rigour** | **Discussion** | **Analysis of the reviewer** |
| --- | --- | --- | --- | --- | --- |
| (Henrickson, Dickson, Mhlanga, & Ludlam, 2015)  Stigma, lack of knowledge and prevalence maintain HIV risk among Black Africans in New Zealand. *Australian and New Zealand Journal of Public Health, 39*(1), 32-37. doi:10.1111/1753-6405.12301 | Journal article | 1.1 **Research**   - Quantitative - Qualitative   **2.Objective or research question**  To explore HIV risks in Black African communities in NZ with a view to informing HIV infection prevention and health promotion programs.  **3. Sample**  **3.1 Data source**  **3.2 Size**  703 (351F; 343M) for quantitative  131 (76F; 54 M) for FGDs  4. **Methods**  Desk estimates of the resident Black African population in New Zealand, and Africans living with HIV;  Anonymous survey administered at African community events and a series of focus groups around the country. | Methodology is consistent with the results | - 1. **Results**   High levels of knowledge and positive attitudes about HIV were more often found in older than younger age groups. Condom use was higher in the younger group than in older age groups. Traditional attitudes still inform some beliefs about HIV. Stigma about HIV and anyone at risk for HIV remains very high among Africans. Western sexual identity constructs are not meaningful.  **1.2 Recommendation**  A culturally informed strategy for risk and stigma reduction is urgently needed. |  |

| **Author, yr, title** | **Type of article** | **Methodology Characteristics of the study** | **Assessing methodological rigour** | **Discussion** | **Analysis of the reviewer** |
| --- | --- | --- | --- | --- | --- |
| (Suphanchaimat et al., 2015)  Challenges in the provision of healthcare services for migrants: a systematic review through providers’ lens. BMC Health Services Research, 15, 390. doi:10.1186/s12913-015-1065-z | Journal article | 1.1 Research  Quantitative  2.Objective or researchquestion  To systematically review the latest literature, which investigated perceptions and attitudes of healthcare providers in managing care for migrants, as well as examining the challenges and barriers faced in their practices.  3. Sample  3.1 Data source  3.2 Size  37 articles  4. Methods  A systematic review | Methodology is consistent with the results | **Results**  language and cultural challenges,  a lack of knowledge of a host country's health system amongst migrant patients.  in-house constraints resulting from heavy workloads and inadequacy of human resources.  Professional norms strongly influenced the behaviours and attitudes of healthcare providers despite conflicting with laws that limited right to health services access for illegal migrants.  **1.2 Recommendation**  Further studies, which explore health care management for migrants in countries with different health insurance models, are recommended. | Successfully  overcoming these barriers like cultural and linguistics dynamics also requires identifying  whether or not the service matched needs of migrant  beneficiaries. |

| **Author, yr, title** | **Type of article** | **Methodology Characteristics of the study** | **Assessing methodological rigour** | **Discussion** | **Analysis of the reviewer** |
| --- | --- | --- | --- | --- | --- |
| (Birukila, Brunton, & Dickson, 2014)  HIV-related risk factors among black African migrants and refugees in Christchurch, New Zealand: results from the Mayisha-NZ survey. *New Zealand Medical Journal 126*(1376), 19-27. | Journal article | 1.1 **Research**  Quantitative  2. **Objective or research question**  To describe the demographic characteristics of, and HIV-related risk behaviours among, black African migrants and refugees in Christchurch.  3. **Sample**  **3.1 Data source**  Participants from 13 different African countries  3.2 **Size**  245 participants (150 men and 95 women) with a mean age of 28 years (range 16 to 58)  4. **Methods**  Cross sectional survey | Methodology is consistent with the results | **Results**  Risk factors for HIV identified:  -low condom use,  -low HIV risk perception,  -having more than one sexual partner,  -previous STI diagnosis and lack of voluntary testing for HIV.  -frequent travels to their countries of origin  **1.2 Recommendation**  To develop an HIV prevention strategy for black Africans in New Zealand that is informed by local evidence. This strategy should also address sexual health needs of Africans including barriers to condom use, the availability of HIV/STI screening services and targeting sexual behaviours that increase vulnerability to HIV infection. | Although there is a high prevalence of HIV amongst black African immigrants, there is evidence to suggest that some Africans are being infected in New Zealand.  •In 2009, when the numbers of heterosexually-acquired HIV cases infected in New Zealand peaked, 7 of the 19 people diagnosed with heterosexually-acquired HIV infected in New Zealand were African according to the AIDS Epidemiology Group (2010).  •The question is by who? The in-born population or other immigrants from other parts of the world?  •This then warrants for an HIV prevention strategy for the whole population despite immigration status or nationality as it is inevitable that sexual mixing across ethnic groups within New Zealand is happening which may result in HIV infection spreading from groups with higher prevalence to the general population. Madlosky and colleagues (2012) concurs that programmes should be designed not to target only specific population groups, but also certain high-burden health problems. This may include treatment and prevention programmes. At the same time, focusing exclusively on interventions which target specific conditions may distract from addressing common ailments and “upstream” determinants of health inequalities, such as socioeconomic status, housing and education |

| **Author, yr, title** | **Type of article** | **Methodology Characteristics of the study** | **Assessing methodological rigour** | **Discussion** | **Analysis of the reviewer** |
| --- | --- | --- | --- | --- | --- |
| (Came, McCreanor, & Rawson, 2016)  The New Zealand Health Strategy 2016: whither health equity? The New Zealand Medical Journal, 129(1447). | Journal article | **1.1 Research**  **Quantitative**  2.**Objective or research question**  To critique the strategy in as it relates to health equity particularly for Māori  **3. Sample**  **3.1 Data source**  **3.2 Size**  One NZ Health strategy  **4. Methods**  Document analysis: Critique of the five NZHS themes of—people powered, closer to home, value and high performance, one team and smart system | Methodology is consistent with the results | **Results**  The NZHS relies on the isolated efforts of committed individuals and organisations to achieve health equity and Te Tiriti engagement, rather than through a planned systems viewpoint  **1.2 Recommendation**  Evidence on health equity and Te Tiriti application suggests efforts need to be sustained, systematic and multi-levelled to be successful, rather than ad hoc and piecemeal. | It is unclear where or how the ‘powered’ part of the term will be applied. Rather use of the term ‘peoplecentred’ or ‘people and whānau-centred’.  Whilst it is noble to employ the collaborative and supportive ethos behind the ‘one team’ theme the Strategy and Roadmap needs to go beyond a narrow definition of health and collaborate with other sectors to achieve wellbeing. This, from a migrant lense could also mean linking the public health system with the panel physcians in sending countries on pre-departure health assessments of migrant and refugees.  Significant challenges that are not included in the Strategy amongst other emerging issues are migrants and refugee health despite the steady fast increase of these groups in New Zealand. Or could it be a case of a diverse range of programmes and projects being implemented by health service providers, social health insurance funds, NGOs, research centres, local governments and other stakeholders in migrant health as mentioned by Came et al., 2016. These stakeholders’ initiatives are not fully reflected in national government policies, meaning that an absence of a blue print policy does not necessarily imply worse health services for migrants in the country in question. |

| **Author, yr, title** | **Type of article** | **Methodology Characteristics of the study** | **Assessing methodological rigour** | **Discussion** | **Analysis of the reviewer** |
| --- | --- | --- | --- | --- | --- |
| (Horner & Ameratunga, 2012)  Monitoring immigrant health and wellbeing in New Zealand: addressing the tyranny of misleading averages. Australian Health Review, 36(4), 390-393. doi:https://doi.org/10.1071/AH11134 | Journal article | 1.1 Research  Quantitative  **2.Objective or research question**  To discuss the 2007 New Zealand’s Settlement National Action Plan and other relevant social policy documents in view of non-existant data and the politicised nature of immigrant research  3. Sample  **3.1 Data source**  2007 New Zealand’s Settlement National Action Plan and other relevant social policy documents.  3.2 Size  **4. Methods**  Desk review | Methodology is consistent with the results | **Results**  Limited or Non-existent data pertaining to immigrant populations across a range of health and wellbeing indicators,  Highly politicised nature of research in this area.  The absence of consistently collected and reported country of birth data in public health settings inhibits the ability of immigrant health and wellbeing advocates to monitor the progress of government and other agencies in achieving the goals of the Action Plan specifically, and social policy more broadly  **1.2 Recommendation**  Routine collection, disaggregation and reporting of country of birth data across the public health system and further investment in research on immigrant populations in New Zealand is recommended | The Auckland Resettlement Strategy 2009-2014 has also identified a lack of reliable information about migrant and refugee population health in the Auckland region nor does it specify interventions to improve data collection |

| **Author, yr, title** | **Type of article** | **Methodology Characteristics of the study** | **Assessing methodological rigour** | **Discussion** | **Analysis of the reviewer** |
| --- | --- | --- | --- | --- | --- |
| (Harding, Seal, Duncan, & Gilmour, 2017)  General practitioner and registrar involvement in refugee health: exploring needs and perceptions. Aust Health Rev. doi:10.1071/AH17093 | Journal article | 1.**1 Research**  Qualitative  2.**Objective or research question**  To identify assess the needs and attitudes of GPs in treating refugees and the perceived effect that refugees have on their practice.  **3. Sample**  **3.1 Data source**  GP and GP Registers  **3.2 Size**  14  **4. Methods**  Indepth interviews | Methodology is consistent with the results | - 1. **Results**   Language, time and culture constraints.  A need for greater information for GPs regarding services available to help manage refugees in rural and regional areas and greater access to those services was demonstrated.  **1.2 Recommendation**  The findings of this study suggest that international medical graduate doctors identified with resettlement problems faced by refugees and may be an important resource for these patients | This study highlights the awareness, empathy and positive attitudes of GPs in regional and rural areas in their approach to treating patients with a refugee background. |

Babar, Z., Pengelly, K., Scahill, S. L., Garg, S., & Shaw, J. (2013). Migrant health in New Zealand: exploring issues concerning medicines access and use. *Journal of Pharmaceutical Health Services Research, 4*(1), 41-49. doi:10.1111/j.1759-8893.2012.00105.x

Benkhalti Jandua, M., Canuto de Medeirosa, B., Bourgeaultb, I., & Tugwell, P. (2015). The inclusion of migrants in health impact assessments: A scoping review. *Environmental Impact Assessment Review, 50*, 16-24.

Birukila, G., Brunton, C., & Dickson, N. (2014). HIV-related risk factors among black African migrants and refugees in Christchurch, New Zealand: results from the Mayisha-NZ survey. *New Zealand Medical Journal 126*(1376), 19-27.

Browne, A. J., Varcoe, C. M., Wong, S. T., Smye, V. L., & Khan, K. B. (2014). Can ethnicity data collected at an organizational level be useful in addressing health and healthcare inequities? *Ethnicity & health, 19*(2), 240-254.

Came, H., McCreanor, T.,

Doole, C.,, & Rawson, E. (2016). The New Zealand Health Strategy 2016: whither health equity? *The New Zealand Medical Journal, 129*(1447).

Choummanivong, C., Poole, G. E., & Cooper, A. (2014). Refugee family reunification and mental health in resettlement. *Kōtuitui: New Zealand Journal of Social Sciences Online, 9*(2), 89-100.

Denholm, J. T., & McBryde, E. S. (2014). Can Australia eliminate TB? Modelling immigration strategies for reaching MDG targets in a low-transmission setting. *Australian and New Zealand Journal of Public Health, 38*(1), 78-82. doi:10.1111/1753-6405.12161

Denholm, J. T., McBryde, E. S., & Brown, G. V. (2012). Ethical evaluation of immigration screening policy for latent tuberculosis infection. *Australian and New Zealand Journal of Public Health, 36*(4), 325-328. doi:10.1111/j.1753-6405.2012.00895.x

Douglas, P., Posey, D., Zenner, D., Robson, J., Abubakar, I., & Giovinazzo, G. (2017). Capacity strengthening through pre-migration tuberculosis screening programmes: IRHWG experiences. *The International Journal of Tuberculosis and Lung Disease, 21*(7), 737-745.

European Centre for Disease Prevention and Control. (2014). *Assessing the burden of key infectious diseases affecting migrant populations in the EU/EEA. Technical Report*. Stockholm: ECDC.

Gray, B., Hilder, J., & Stubbe, M. (2012). How to use interpreters in general practice: the development of a New Zealand toolkit. *Journal of Primary Health Care, 4*(1), 52-61. doi:https://doi.org/10.1071/HC12052

Harding, C., Seal, A., Duncan, G., & Gilmour, A. (2017). General practitioner and registrar involvement in refugee health: exploring needs and perceptions. *Aust Health Rev*. doi:10.1071/AH17093

Henrickson, M., Dickson, N., Mhlanga, F., & Ludlam, A. (2015). Stigma, lack of knowledge and prevalence maintain HIV risk among Black Africans in New Zealand. *Australian and New Zealand Journal of Public Health, 39*(1), 32-37. doi:10.1111/1753-6405.12301

Horner, J., & Ameratunga, S. N. (2012). Monitoring immigrant health and wellbeing in New Zealand: addressing the tyranny of misleading averages. *Australian Health Review, 36*(4), 390-393. doi:https://doi.org/10.1071/AH11134

Jackson, Y., Pinto, A., & Pett, S. (2014). Chagas disease in Australia and New Zealand: risks and needs for public health interventions. *Tropical Medicine & International Health, 19*(2), 212-218. doi:10.1111/tmi.12235

Marlowe, J., & Elliott, S. (2014). Global trends and refugee settlement in New Zealand. *Kōtuitui: New Zealand Journal of Social Sciences Online, 9*(2), 43-49. doi:https://doi-org.ezproxy.aut.ac.nz/10.1080/1177083X.2014.953186

Marlowe, J. M., Bartley, A., & Hibtit, A. (2014). The New Zealand Refugee Resettlement Strategy: implications for identity, acculturation and civic participation. *Kotuitui: New Zealand Journal of Social Sciences, 9*(2), 60-69.

Martinez, O., Wu, E., Sandfort, T., Dodge, B., Carballo-Dieguez, A., Pinto, R., . . . Chavez-Baray, S. (2015). Evaluating the Impact of Immigration Policies on Health Status Among Undocumented Immigrants: A Systematic Review [journal article]. *Journal of Immigrant and Minority Health, 17*(3), 947-970. doi:10.1007/s10903-013-9968-4

Mette Fløe Hvass, A., & Wejse, C. (2017). Systematic health screening of refugees after resettlement in recipient countries: A scoping review. *Annals of Human Biology, 44*(5).

Mladovsky, P., Rechel, B., Ingleby, D., & McKee, M. (2012). Responding to diversity: An exploratory study of migrant health policies in Europe [Review]. *Health Policy, 105*(1), 1-9. doi:10.1016/j.healthpol.2012.01.007

Morgan, R. K. (2012). Institutionalising health impact assessment:the New Zealand experience. *Impact Assessment and Project Appraisal, 26*(1), 2-16.

Pareek, M., Greenaway, C., Noori, T., Munoz, J., & Zenner, D. (2016). The impact of migration on tuberculosis epidemiology and control in high-income countries: a review [journal article]. *BMC Medicine, 14*(1), 48. doi:10.1186/s12916-016-0595-5

Perumal, L. (2011). *Health Needs Assessments for Middle East, Latin America and African people living in Auckland Region*. Auckland: Auckland District Health Board.

Reid, A. (2012). Under-use of migrants' employment skills linked to poorer mental health. *Australian and New Zealand Journal of Public Health, 36*(2), 120-125. doi:10.1111/j.1753-6405.2012.00842.x

Rungan, S., Reeve, A. M., Reed, P. W., & Voss, L. (2013). Health Needs of Refugee Children Younger Than 5 Years Arriving in New Zealand. *The Pediatric Infectious Disease Journal, 32*(12), e432-e436. doi:10.1097/INF.0b013e3182a11526

Shrestha-Ranjit , J., Patterson, E., Manias, E., Payne, D., & Koziol-McLain, J. (2017). Effectiveness of Primary Health Care Services in Addressing Mental Health Needs of Minority Refugee Population in New Zealand. *Mental Health Nursing, 38*(4).

Suphanchaimat, R., Kantamaturapoj, K., Putthasri, W., & Prakongsai, P. (2015). Challenges in the provision of healthcare services for migrants: a systematic review through providers’ lens. *BMC Health Services Research, 15*, 390. doi:10.1186/s12913-015-1065-z

White, Z., Painter, J., Douglas, P., Abubakar, I., Njoo, H., Archibald, C., . . . Posey, D. L. (2017). Immigrant Arrival and Tuberculosis among Large Immigrant- and Refugee-Receiving Countries, 2005–2009. *Tuberculosis Research and Treatment, 2017*, 8567893. doi:10.1155/2017/8567893

Wickramage, K., & Mosca, D. (2014). Can Migration Health Assessments Become a Mechanism for Global Public Health Good? *International Journal of Environmental Research and Public Health, 11*(10), 9954-9963. doi:10.3390/ijerph111009954
